# Two new species of *Limbodessus* diving beetles from New Guinea - short verbal descriptions flanked by online content (digital photography, μCT scans, drawings and DNA sequence data)

**DOI:** 10.3897/BDJ.3.e7096

**Published:** 2015-12-22

**Authors:** Michael Balke, Bernhard Ruthensteiner, Evie Lilly Warikar, Katja Neven, Lars Hendrich

**Affiliations:** ‡ Zoologische Staatssammlung, Munich, Germany; § Department of Biology, Universitas Cendrawasih, Jayapura, Indonesia

**Keywords:** Dytiscidae, *
Limbodessus
*, new species, high resolution photography, μCT scans, DNA sequence data, minimalistic descriptions, New Guinea

## Abstract

**Background:**

To date only one species of *Limbodessus* diving beetles has been reported from the Island of New Guinea, *L. compactus* (Clark, 1862), which is widerspread in the Australian region.

**New information:**

We describe two new species of microendemic New Guinea *Limbodessus* and use a compact descriptive format flanked by enriched online content in wiki powered species pages. *Limbodessus baliem* **sp.n.** is described from ca. 1,600 m altitude in the Baliem Valley of Papua and *Limbodessus alexanderi* **sp.n.** from >3,000 m altitude north of Sugapa, Papua.

Based on our analysis, we also transfer three species from other genera to *Limbodessus* Guignot, 1939, with the following changes: *Limbodessus deflectus* (Ordish, 1966), **new combination**; *Limbodessus leveri* (J. Balfour-Browne, 1944), **new combination**; and *Limbodessus plicatus* (Sharp, 1882), **new combination**.

## Introduction

*Limbodessus* Guignot (1939) contains 71 described species mostly distributed in the Australasian region, few species are Oriental or reach the Palearctic in Japan (Nilsson (2001), Nilsson (2015). The majority of species was described from underground waters in Australia, the otherwise epigean species prefer stagnant water habitats or densely vegetated backflows or marginal areas of slowly flowing streams. Underground and interstitial species show adaptations to their environment, such as reduction or loss of the eyes as well as reduced wings (Watts and Humphreys (1999), Larson (1994). Based on their morphology being different from the epigean species (Watts and Leijs (2005), new genera such as *Boongurrus* Larson and *Tjirtudessus* Watts & Humphreys were suggested, but later synonymised with *Limbodessus* by Balke and Ribera (2004) based on molecular phylogenetic data and a morphological apomorphy (male paramere with hook or bent finger like apical part).

Here we describe two new microendemic species from New Guinea. One occurs in an intramontane depression at 1,600–1,700 m and the other one in the tropical-montane to alpine habitat above 3,000 m. We utilize a compact, integrative descriptive format following Riedel et al. (2013a), Riedel et al. (2013b) combining morphological and molecular evidence. We provide high resolution online digital imaging resources, as well as μCT data illustrating characters not explicitly described here but anticipating potential interest in certain structures in the future (e.g. sculpture of metacoxa, width and structure of lateral wings of metaventrite).

## Materials and methods

Specimens are in the following collections:

**CLH** Collection of Lars Hendrich, Munich, Germany (property of NHMW)

**MZB** Museum Zoologicum Bogoriense, now LIPI RC Biology, Division of Zoology, Cibinong, West Java, Indonesia

**NHMW** Naturhistorisches Museum Wien, Vienna, Austria

**NMPC** National Museum, Prague, Czech Republic

**ZSM** Zoologische Staatsammlung München, Munich, Germany

Measurements were taken with a Leica M205 C stereomicroscope. The following abbreviations were used: TL (total body length), TL-H (total body length without head), and MW (maximum body width). UNCEN legit indicates specimens collected during a field course with the Cendrawasih University, Jayapura, Papua (UNCEN).

DNA sequence data were generated using standard methods described in detail in our laboratory wiki: http://zsm-entomology.de/wiki/The_Beetle_D_N_A_Lab

Digital images were taken with a Nikon D3X equipped with a bellow or expansion rings and lenses: Mitutoyo 10x ELWD Planapo or Leitz Photar 25/2.8. Illumination came from three compact Nikon flashes, and the instrument was moved on an Isel linear drive or Cognisys Stackshot (for very small steps 1–7 μm). Image stacks were combined using the method A in Helicon focus software.

For micro-CT scanning, the specimens were attached with soft dental wax to tips of glass Pasteur pipettes. Scanning was performed with a Phoenix Nanotom M (GE Measurement & Control, Wunstorf, Germany) cone beam CT scanner at a voltage of 60 kV and a current of 240mA (*Limbodessus baliem*, *Kaef2-gr*) or a voltage of 50 kV and a current of 275 mA (*Kaef2-kl*) respectively, using a molybdenum target. Each scan took 144 minutes. 1.440 projections were prepared per scan. The 3D datasets (prepared with the datos|x reconstruction software, GE Measurement & Control) were examined by volume rendering with Drishti 2.3.2 Limaye (2012) and VGStudioMax 2.2 (Visual Graphics GmbH, Heidelberg, Germany) software.

In addition, surface meshes were generated using the threshold tool in the segmentation editor of the software Amira 5.4.5 (FEI VisualizationSciences Group, Burlington MA, USA). For varying intensities in the volume data, the threshold was slightly locally adjusted. The attachment wax, which has X-ray absorption nearly as intense as that of the specimens’ skeletons, was removed during segmentation by a combination of threshold and manual (lasso and brush tools) segmentation. To constrain total mesh complexity (eventually to ca. one million faces) and reduce file size to a tolerable level, all internal structures were removed.

The PDF 3D models were prepared largely following the procedures outlined by Ruthensteiner and Heß (2008).

## Data resources

DNA sequences were uploaded to EMBL and are available under accession numbers LN884305–LN884314.

The cox1 sequences are available in alignment format on Dryad: doi:10.5061/dryad.q1b24. Newly generated data are appended here in fasta format Suppl. material 1.

Animated videos of the μCT scans are available at YouTube tagged as "Limbodessus". The μCT data were deposited in the Morphosource database in their own project.

## Taxon treatments

### Limbodessus alexanderi

Balke & Hendrich sp. n.

urn:lsid:zoobank.org:act:9C940C82-646F-4075-86B9-3E88D397ABEA

#### Materials

- **Type status:** Holotype. **Occurrence:** recordedBy: Riedel; sex: male; lifeStage: adult; **Taxon:** taxonID: urn:lsid:zoobank.org:act:9C940C82-646F-4075-86B9-3E88D397ABEA; scientificName: Limbodessus alexanderi; order: Coleoptera; family: Dytiscidae; **Location:** island: New Guinea; country: Indonesia; stateProvince: Papua; locality: N Sugapa; verbatimElevation: 3000 m; verbatimCoordinates: 3°40'11.90"S, 137° 4'51.31"E; decimalLatitude: -3.669972; decimalLongitude: 137.080919; **Event:** samplingProtocol: collected with strainer; eventDate: 27.xii.1995; **Record Level:** institutionCode: NHMW; collectionCode: Insects
- **Type status:** Paratype. **Occurrence:** recordedBy: Riedel; individualID: four of them with green extraction voucher labels: M. Balke 4424, 4425, 6423, 6424; individualCount: 130; lifeStage: adult; **Taxon:** taxonID: urn:lsid:zoobank.org:act:9C940C82-646F-4075-86B9-3E88D397ABEA; scientificName: Limbodessus alexanderi; order: Coleoptera; family: Dytiscidae; **Location:** island: New Guinea; country: Indonesia; stateProvince: Papua; locality: N Sugapa; verbatimElevation: 3000 m; verbatimCoordinates: 3°40'11.90"S, 137° 4'51.31"E; decimalLatitude: -3.669972; decimalLongitude: 137.080919; **Event:** samplingProtocol: collected with strainer; eventDate: 27.xii.1995; **Record Level:** institutionCode: MZB, NHMW, ZSM; collectionCode: Insects

#### Description

**Figure 1.**
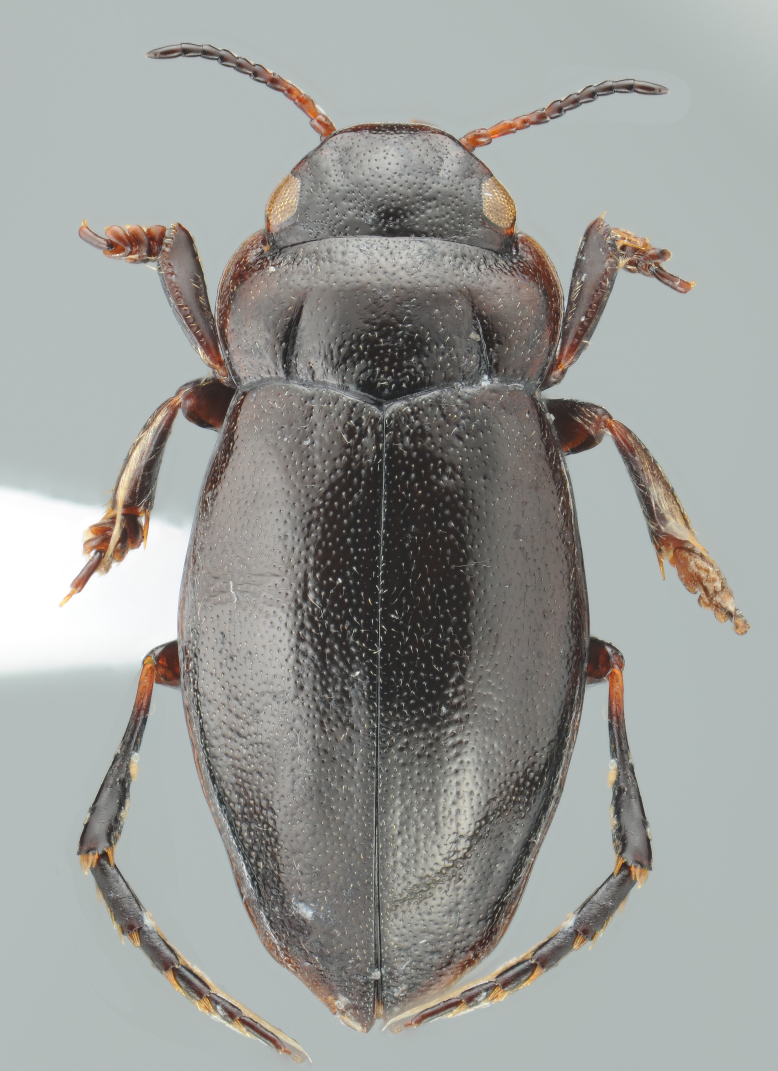
*Limbodessus alexanderi*, male, dorsal habitus. Length of beetle: 3.1 mm.

**Figure 2.**
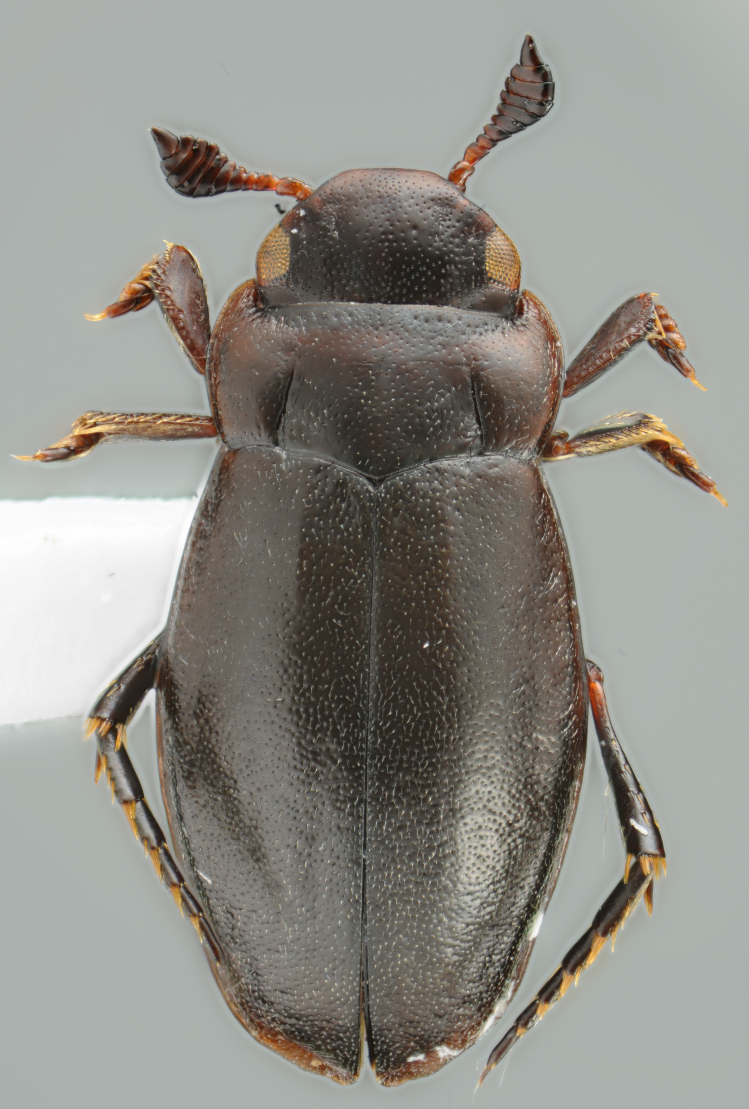
*Limbodessus alexanderi*, female, dorsal habitus. Length of beetle: 3.1 mm.

**Figure 3.**
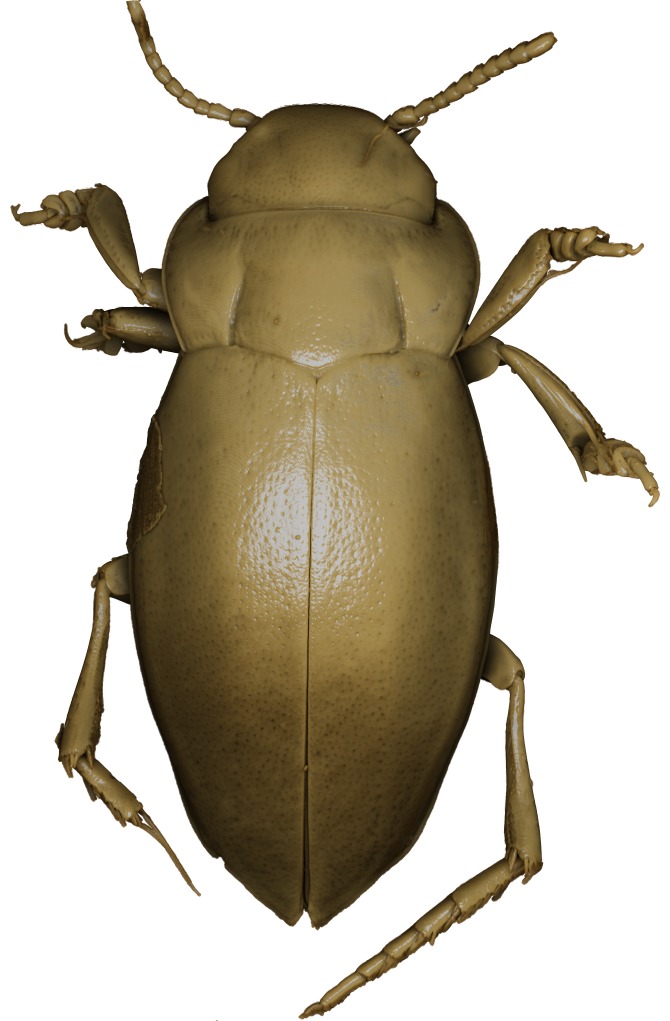
*Limbodessus alexanderi*, male, dorsal habitus. μCT scan. Length of beetle: 3.1 mm.

**Figure 4.**
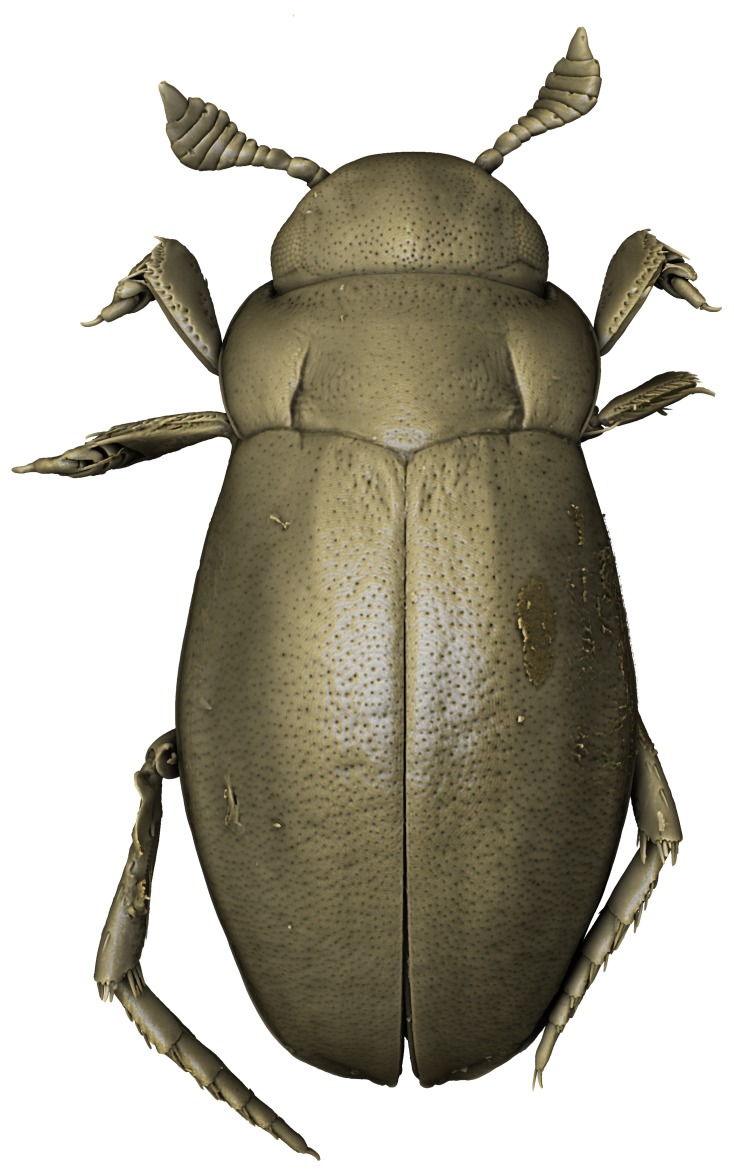
*Limbodessus alexanderi*, female, dorsal habitus. μCT scan. Length of beetle: 3.1 mm.

**Figure 5.**
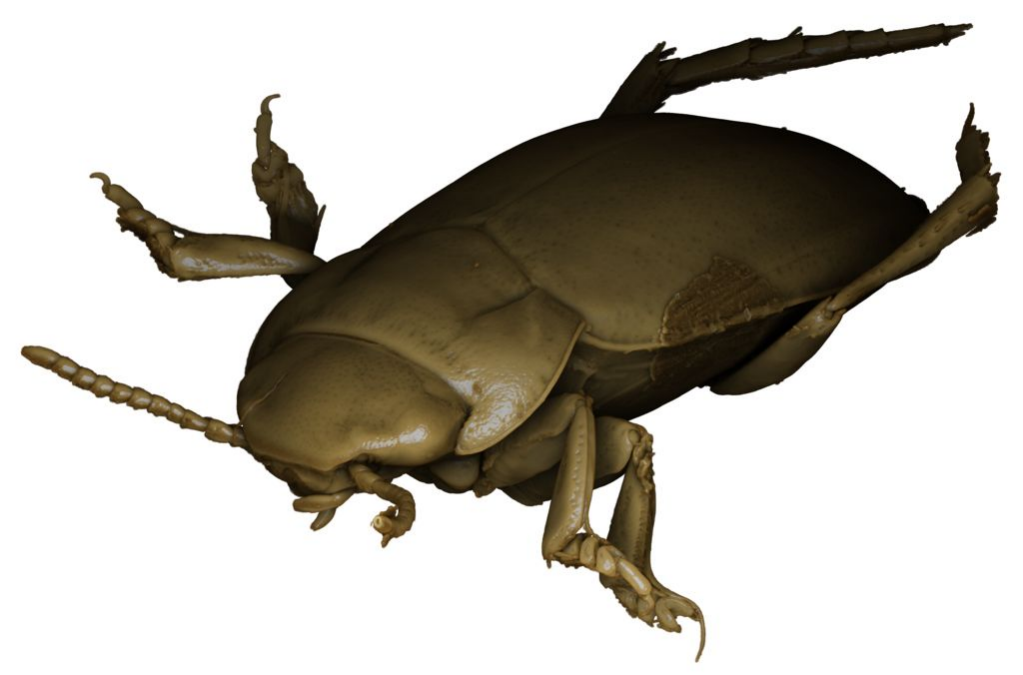
*Limbodessus alexanderi*, male, frontal habitus. μCT scan. Length of beetle: 3.1 mm.

**Figure 6.**
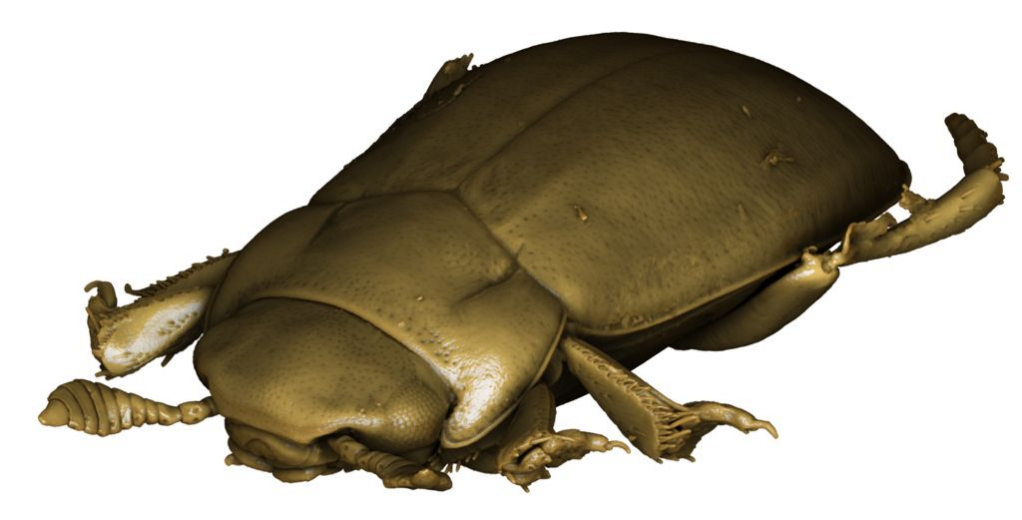
*Limbodessus alexanderi*, female, frontal habitus. μCT scan. Length of beetle: 3.1 mm.

**Figure 7.**
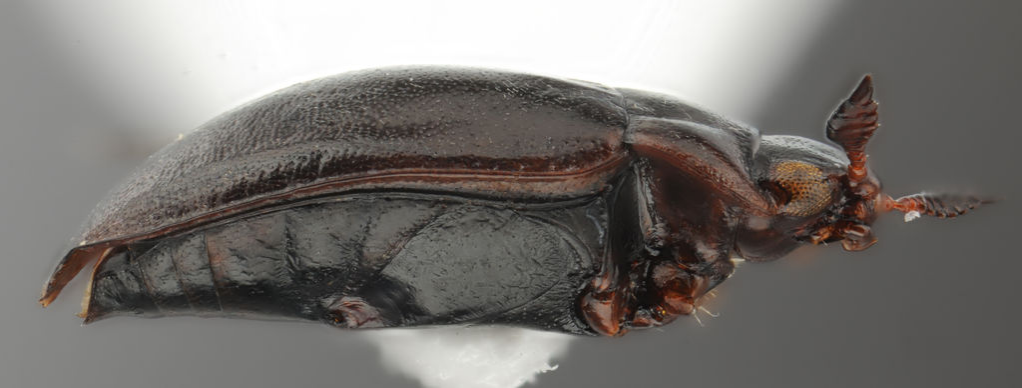
*Limbodessus alexanderi*, female, lateral habitus. Length of beetle: 3.1 mm.

**Figure 8.**
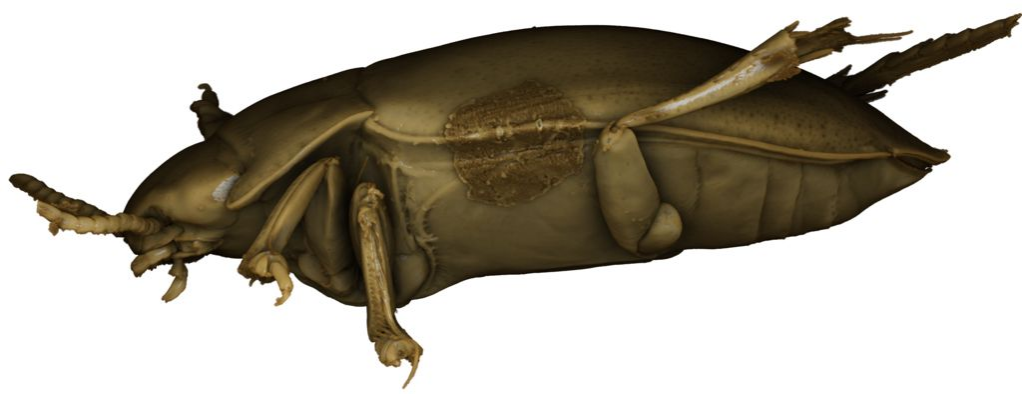
*Limbodessus alexanderi*, male, lateral habitus. μCT scan. Length of beetle: 3.1 mm.

A large, dark brown to black *Limbodessus*: length of body 3.0–3.5 mm (N=20); with pronounced habitus disruption between pronotum and elytron Figs 1, 2, 3, 4, 5, 6, 37, 39; dorsoventrally rather thick Figs 7, 8; cervical line present but faint in male or partly dissolved into punctures in females, distinct pronotal plica present, elytral plica absent; flight wings vestigal; metacoxa and metaventrite with few punctures only Fig. 22​; ventral side with notable modifications: elytral epipleuron apically modified: slightly concave, with inner margin dilated, its form reminding of a spear tip or shallow spoon Figs 7, 16, 17, 37, 39​.

Sexes dimorphic, see below.

##### Male

Antenna filiform Figs 1, 3, 5. Dorsal surface with coarse punctures but otherwise with shiny surface. Median lobe of aedeagus as in Figs 25, 26 with tiny setae on tip, paramere as in Fig. 31.

##### Female

Antenna with strongly enlarged antennomeres forming a conspicuous club Figs 2, 4, 6, 17. Dorsal surface with very fine microreticulation between surface punctures and surfaces therefore appearing matt Figs 2, 10​.

#### Etymology

Named after Alexander Riedel who discovered this species. The species name is a noun in the genitive case.

#### Distribution

Indonesian New Guinea, known only from the type locality which is the mountain range north of Sugapa, Papua (Fig. 23​).

#### Ecology

Puddles in high altitude grassland in the tropical montane / subalpine habitat. Here, a second, much smaller and black *Limbodessus* was also collected which is the female of an undescribed species.

#### Conservation

The species is most likely not threatened due to its occurrence on remote high altitude plateau.

#### Online resources

Higher resolution digital images, μCT data as well as sequence data have been deposited in public databases. The Species-ID species page is a versioned wiki site and can be enhanced through community contributions Hendrich and Balke (2011).

##### Species-ID species page

http://species-id.net/wiki/Limbodessus_alexanderi

##### DNA sequences

Partial 3' *cox1* sequence deposited at: LN884309–LN884312.

##### μCT data

In two Morphosource projects here and here.

### Limbodessus baliem

Balke & Hendrich sp. n.

urn:lsid:zoobank.org:act:3EC40F14-C7D9-42C8-84EB-91ADF93C9F7D

#### Materials

- **Type status:** Holotype. **Occurrence:** recordNumber: IR1; recordedBy: Balke & Hendrich; sex: male; lifeStage: adult; **Taxon:** taxonID: urn:lsid:zoobank.org:act:3EC40F14-C7D9-42C8-84EB-91ADF93C9F7D; scientificName: Limbodessus baliem; order: Coleoptera; family: Dytiscidae; **Location:** island: New Guinea; country: Indonesia; stateProvince: Papua; locality: Wamena; verbatimElevation: 1600 m; locationRemarks: water holes near runway; verbatimCoordinates: 4° 6'16.76"S, 138°57'37.96"E; **Event:** samplingProtocol: collected with strainer; eventDate: 31.viii. & 6.ix.1990; **Record Level:** institutionCode: NHMW; collectionCode: Insects
- **Type status:** Paratype. **Occurrence:** recordNumber: IR1; recordedBy: Balke & Hendrich; individualCount: 15; lifeStage: adult; **Taxon:** taxonID: urn:lsid:zoobank.org:act:3EC40F14-C7D9-42C8-84EB-91ADF93C9F7D; scientificName: Limbodessus baliem; order: Coleoptera; family: Dytiscidae; **Location:** island: New Guinea; country: Indonesia; stateProvince: Papua; locality: Wamena; verbatimElevation: 1600 m; locationRemarks: water holes near runway; verbatimCoordinates: 4° 6'16.76"S, 138°57'37.96"E; **Event:** samplingProtocol: collected with strainer; eventDate: 31.viii. & 6.ix.1990; **Record Level:** institutionCode: NHMW; collectionCode: Insects
- **Type status:** Paratype. **Occurrence:** recordNumber: IR54A = 57; recordedBy: Balke; individualCount: 3; lifeStage: adult; **Taxon:** taxonID: urn:lsid:zoobank.org:act:3EC40F14-C7D9-42C8-84EB-91ADF93C9F7D; scientificName: Limbodessus baliem; order: Coleoptera; family: Dytiscidae; **Location:** island: New Guinea; country: Indonesia; stateProvince: Papua; locality: Wamena; verbatimElevation: 1700 m; locationRemarks: water holes near runway; verbatimCoordinates: 4° 6'16.76"S, 138°57'37.96"E; **Event:** samplingProtocol: collected with strainer; eventDate: 20.-27.ix.1992; **Record Level:** institutionCode: NHMW; collectionCode: Insects
- **Type status:** Paratype. **Occurrence:** recordedBy: Riedel; individualCount: 1; lifeStage: adult; **Taxon:** taxonID: urn:lsid:zoobank.org:act:3EC40F14-C7D9-42C8-84EB-91ADF93C9F7D; scientificName: Limbodessus baliem; order: Coleoptera; family: Dytiscidae; **Location:** island: New Guinea; country: Indonesia; stateProvince: Papua; locality: Wamena; verbatimElevation: 1700 m; locationRemarks: water holes near runway; verbatimCoordinates: 4° 6'16.76"S, 138°57'37.96"E; **Event:** samplingProtocol: collected with strainer; eventDate: 15.x.1993; **Record Level:** institutionCode: ZSM; collectionCode: Insects
- **Type status:** Paratype. **Occurrence:** recordNumber: IR52; recordedBy: Balke; individualCount: 39; lifeStage: adult; **Taxon:** taxonID: urn:lsid:zoobank.org:act:3EC40F14-C7D9-42C8-84EB-91ADF93C9F7D; scientificName: Limbodessus baliem; order: Coleoptera; family: Dytiscidae; **Location:** island: New Guinea; country: Indonesia; stateProvince: Papua; locality: Wamena; verbatimElevation: 1600 m; locationRemarks: water holes near runway; verbatimCoordinates: 4° 6'16.76"S, 138°57'37.96"E; **Event:** samplingProtocol: collected with strainer; eventDate: 19.ix.1992; **Record Level:** institutionCode: NHMW; collectionCode: Insects
- **Type status:** Paratype. **Occurrence:** recordNumber: IR53; recordedBy: Balke; individualCount: 3; lifeStage: adult; **Taxon:** taxonID: urn:lsid:zoobank.org:act:3EC40F14-C7D9-42C8-84EB-91ADF93C9F7D; scientificName: Limbodessus baliem; order: Coleoptera; family: Dytiscidae; **Location:** island: New Guinea; country: Indonesia; stateProvince: Papua; locality: Wamena; verbatimElevation: 1600 m; locationRemarks: water holes near runway; verbatimCoordinates: 4° 6'16.76"S, 138°57'37.96"E; **Event:** samplingProtocol: collected with strainer; eventDate: 19.ix.1992; **Record Level:** institutionCode: NHMW; collectionCode: Insects
- **Type status:** Paratype. **Occurrence:** recordNumber: PAP04; recordedBy: UNCEN; individualID: one of them with green extraction voucher label M. Balke 5082 and one with 5083; individualCount: 37; lifeStage: adult; **Taxon:** taxonID: urn:lsid:zoobank.org:act:3EC40F14-C7D9-42C8-84EB-91ADF93C9F7D; scientificName: Limbodessus baliem; order: Coleoptera; family: Dytiscidae; **Location:** island: New Guinea; country: Indonesia; stateProvince: Papua; locality: Wamena; verbatimElevation: 1600 m; locationRemarks: water holes near runway; verbatimCoordinates: 04 06.323S, 138 57.693E; **Event:** samplingProtocol: collected with strainer; eventDate: 18.x.2011; **Record Level:** institutionCode: MZB, ZSM; collectionCode: Insects
- **Type status:** Paratype. **Occurrence:** recordNumber: PAP05; recordedBy: UNCEN; individualCount: 10; lifeStage: adult; **Taxon:** taxonID: urn:lsid:zoobank.org:act:3EC40F14-C7D9-42C8-84EB-91ADF93C9F7D; scientificName: Limbodessus baliem; order: Coleoptera; family: Dytiscidae; **Location:** island: New Guinea; country: Indonesia; stateProvince: Papua; locality: Wamena, 20 mins towards Jiwika; verbatimElevation: 1620 m; locationRemarks: limestone creek; verbatimCoordinates: 03 56.953S, 138 54.375E; **Event:** samplingProtocol: collected with strainer; eventDate: 18.x.2011; **Record Level:** institutionCode: MZB, ZSM; collectionCode: Insects
- **Type status:** Paratype. **Occurrence:** recordedBy: J. Hájek & J. Šumpich; individualCount: 8; lifeStage: adult; **Taxon:** taxonID: urn:lsid:zoobank.org:act:3EC40F14-C7D9-42C8-84EB-91ADF93C9F7D; scientificName: Limbodessus baliem; order: Coleoptera; family: Dytiscidae; **Location:** island: New Guinea; country: Indonesia; stateProvince: Papua; locality: Jiwika; verbatimLocality: INDONESIA, Papua: Jayawijaya Distr., Baliem valley, 14 km NNE of Wamena, wetland & gardens nr Jiwika [Kurulu], 03°58.0-5'S, 138°55.8 56.3'E; 1660 m, J.Hájek & J.Šumpich leg., 6.ii.2015; verbatimElevation: 1660 m; locationRemarks: wetland; verbatimCoordinates: 03°58.0'S, 138°55.8'E; **Event:** samplingProtocol: collected with strainer; eventDate: 6.ii.2015; **Record Level:** institutionCode: NMPC; collectionCode: Insects
- **Type status:** Paratype. **Occurrence:** recordedBy: J. Hájek & J. Šumpich; individualCount: 31; lifeStage: adult; **Taxon:** taxonID: urn:lsid:zoobank.org:act:3EC40F14-C7D9-42C8-84EB-91ADF93C9F7D; scientificName: Limbodessus baliem; order: Coleoptera; family: Dytiscidae; **Location:** island: New Guinea; country: Indonesia; stateProvince: Papua; locality: Wamena; verbatimLocality: INDONESIA, Papua: Jayawijaya Distr., Baliem valley, Wamena env., ruderal & open marsh nr Baliem river, 04°04.9'S, 138°57.3'E; 1650 m, J.Hájek & J.Šumpich leg., 31.i.+8.ii.2015; verbatimElevation: 1650 m; locationRemarks: wetland; verbatimCoordinates: 04°04.9'S, 138°57.3'E; **Event:** samplingProtocol: collected with strainer; eventDate: 31.i.+8.ii.2015; **Record Level:** institutionCode: NMPC; collectionCode: Insects

#### Description

**Figure 9.**
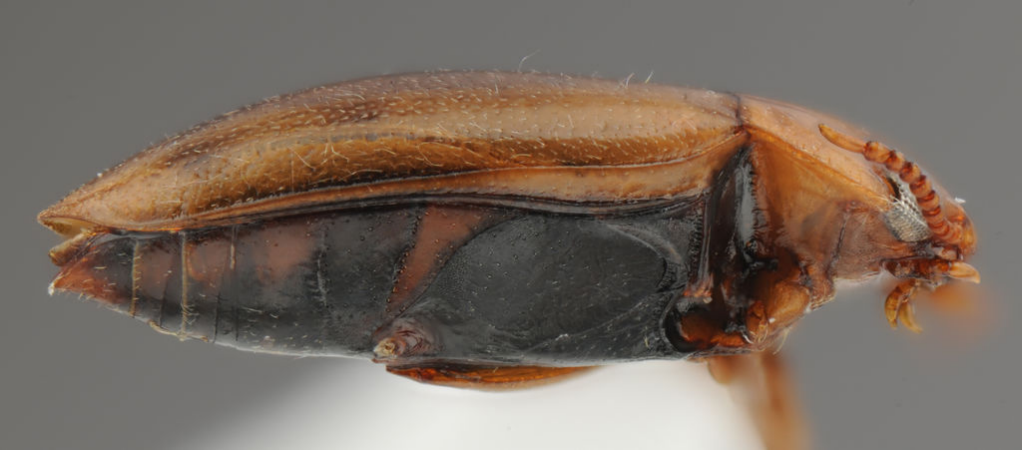
*Limbodessus baliem*, female, lateral habitus. Length of beetle: 2.8 mm.

**Figure 10.**
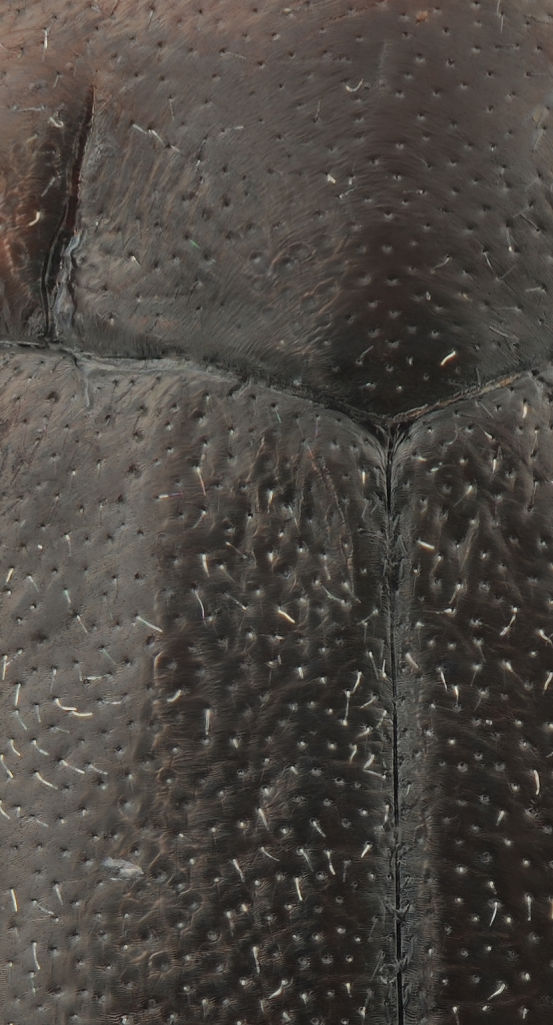
*Limbodessus alexanderi*, female, detailed elytral and pronotal sculpture. Lens: Mitutoyo ELWD 10x.

**Figure 11.**
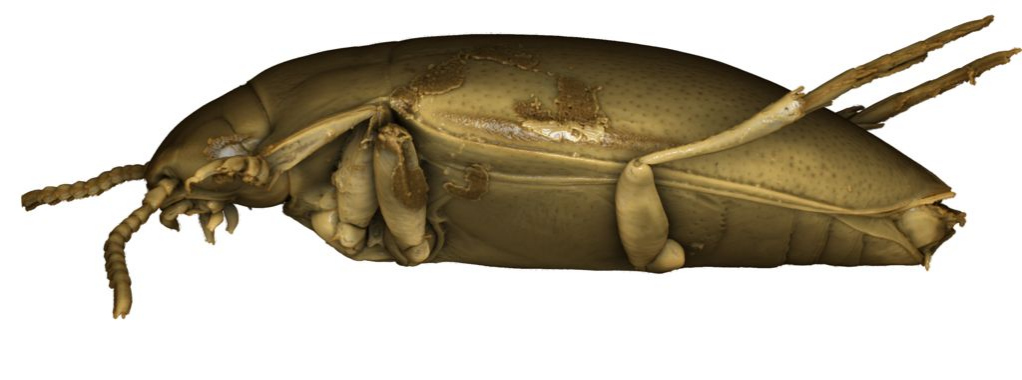
*Limbodessus baliem*, male, lateral habitus. μCT scan. Length of beetle: 2.8 mm.

A large, mainly yellow to orange *Limbodessus*: length of body 2.6–3.0 mm (N=20); with slight habitus disruption between pronotum and elytron Figs 13, 14, 15, 18; dorso ventrally rather flattened Figs 9, 11; cervical line present (sometimes faint or partly dissolved into punctures in females), distinct pronotal and elytral plicae present Figs 13, 14, 15, 18, 41; fully developed flight wings present. Metacoxa and metaventrite with few punctures only Fig. 27​. Ventral side without modified elytral epipleuron.

Sexes dimorphic, see below.

##### Male

Antenna filiform Figs 13, 15, 18, 21, 24​. Dorsal surface with coarse punctures but otherwise with shiny surface Figs 13, 19. Ventrally shiny, dark orange to dark brown Fig. 21​. Median lobe of aedeagus as in Figs 29, 30 with tiny setae on tip, paramere as in Fig. 34.

##### Female

**Figure 12.**
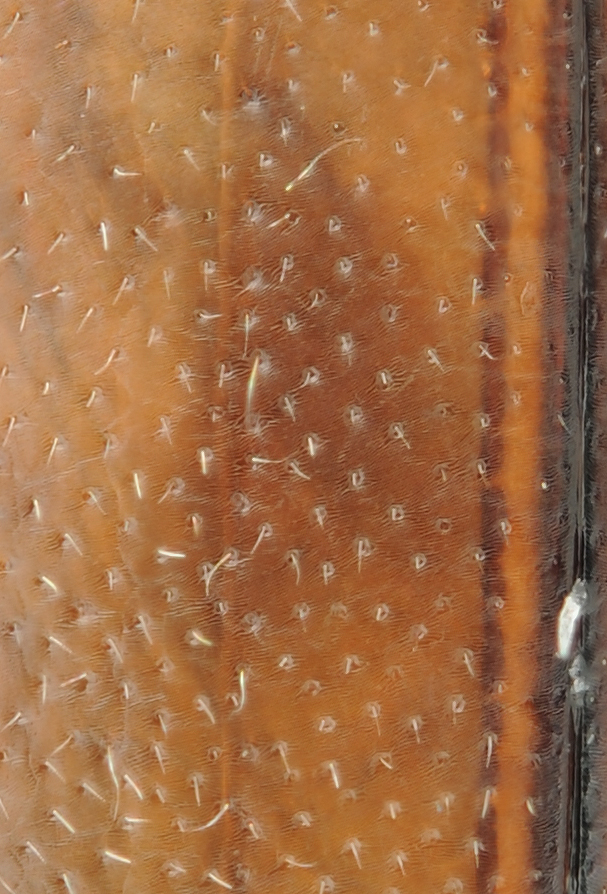
*Limbodessus baliem*, female, detailed elytral and pronotal sculpture. Lens: Mitutoyo ELWD 10x.

**Figure 13.**
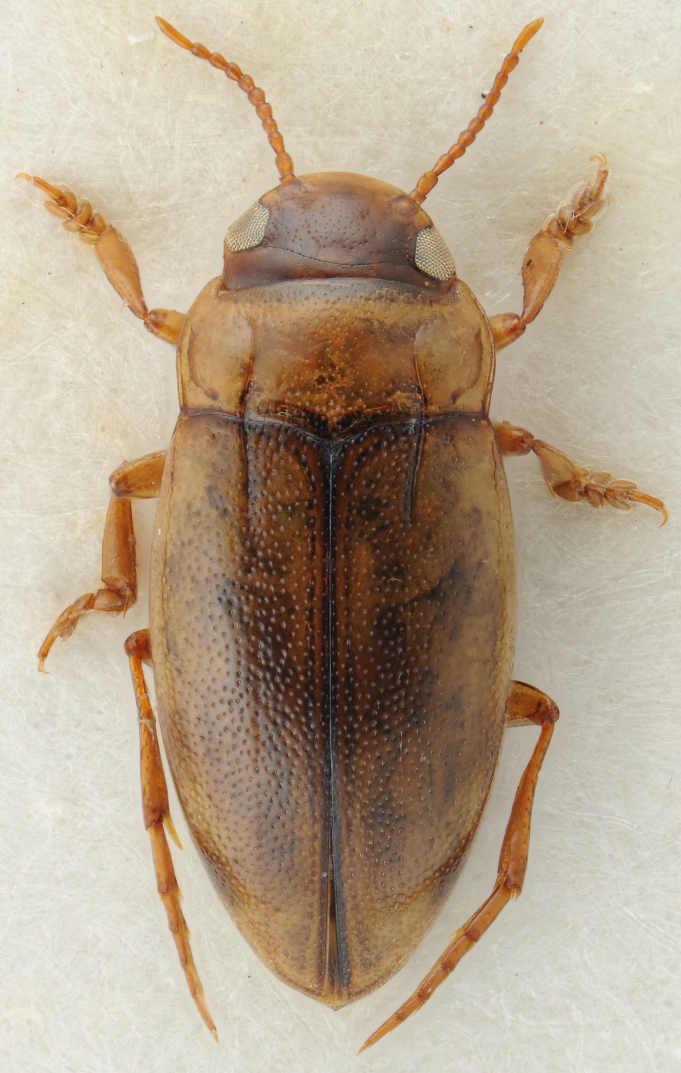
*Limbodessus baliem*, male, habitus dorsally. Length of beetle: 2.8 mm.

**Figure 14.**
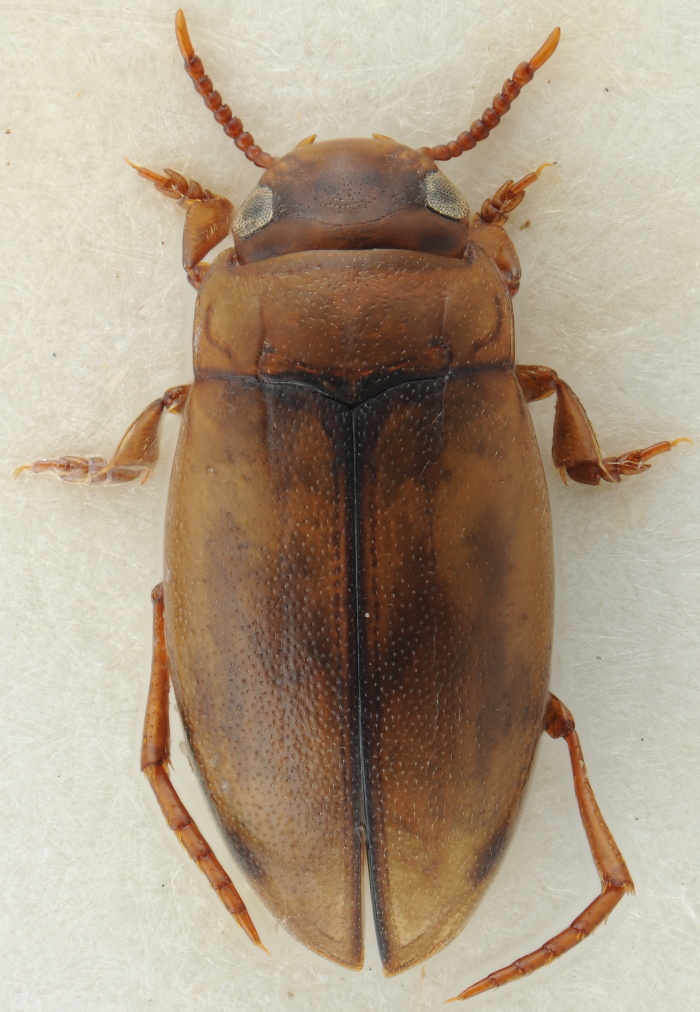
*Limbodessus baliem*, female, habitus dorsally. Length of beetle: 2.8 mm.

**Figure 15.**
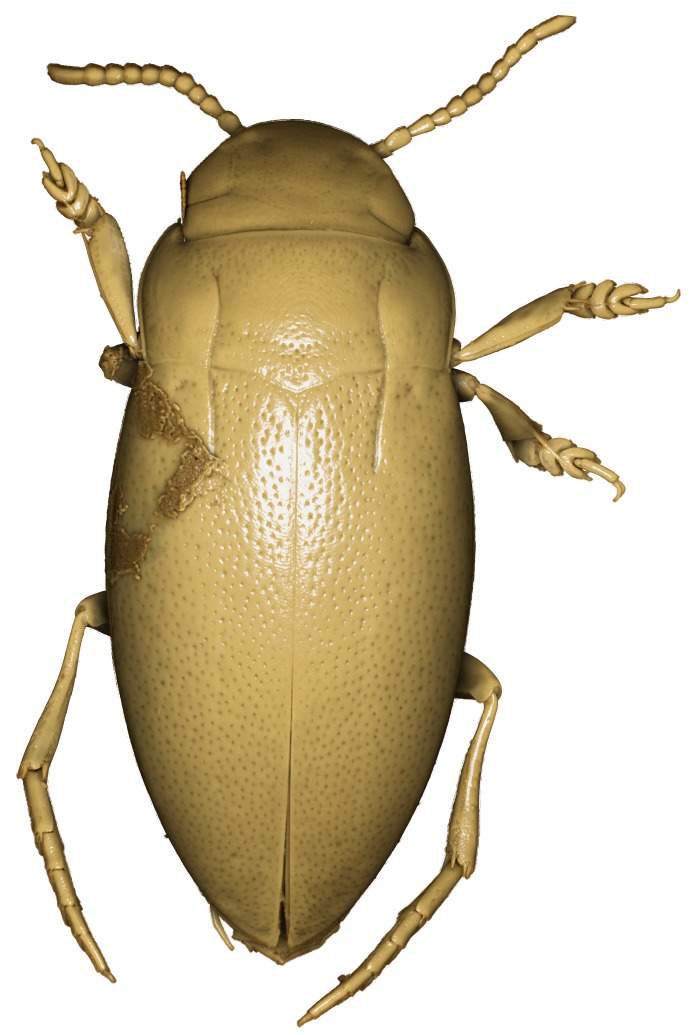
*Limbodessus baliem*, male, habitus dorsally. μCT scan. Length of beetle: 2.8 mm.

**Figure 16.**
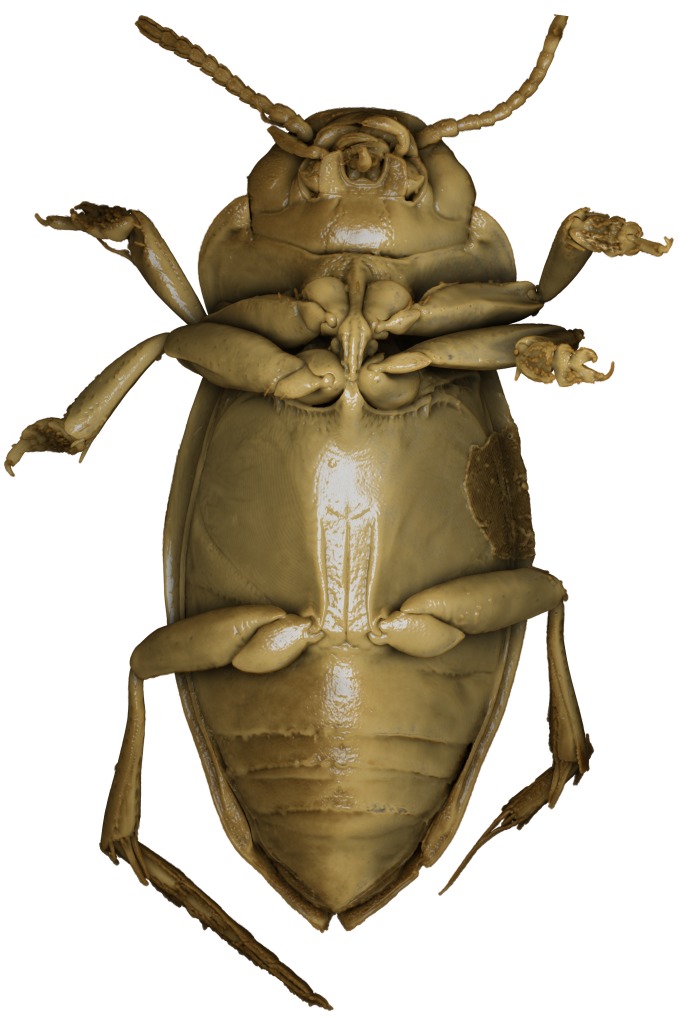
*Limbodessus alexanderi*, male, habitus ventrally. μCT scan. Length of beetle: 3.1 mm.

**Figure 17.**
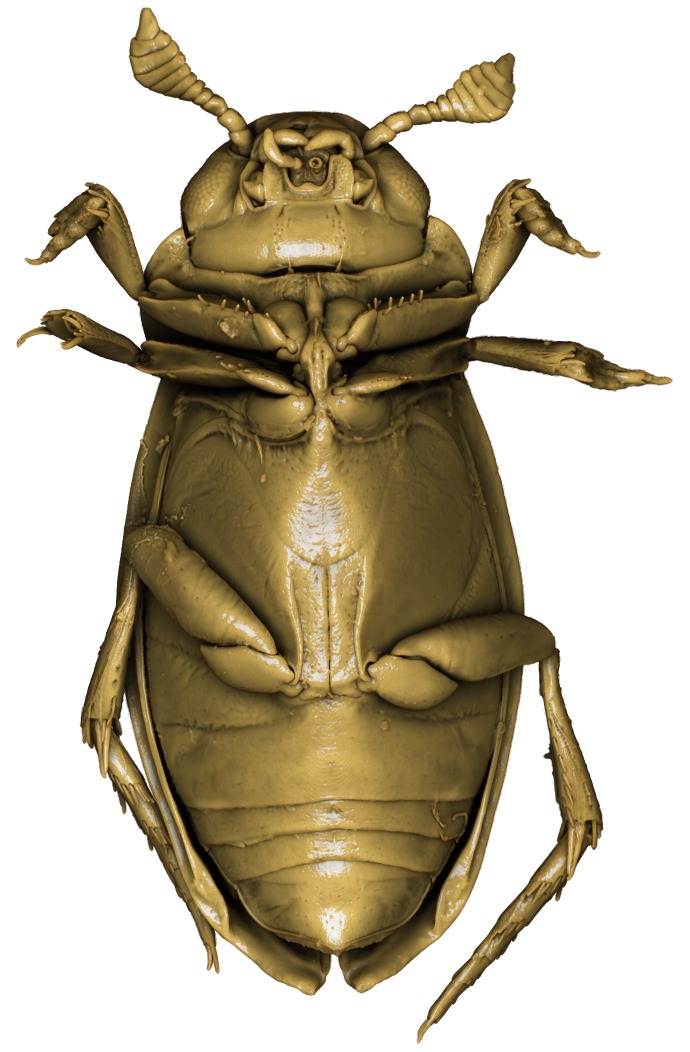
*Limbodessus alexanderi*, female, habitus ventrally. μCT scan. Length of beetle: 3.1 mm.

**Figure 18.**
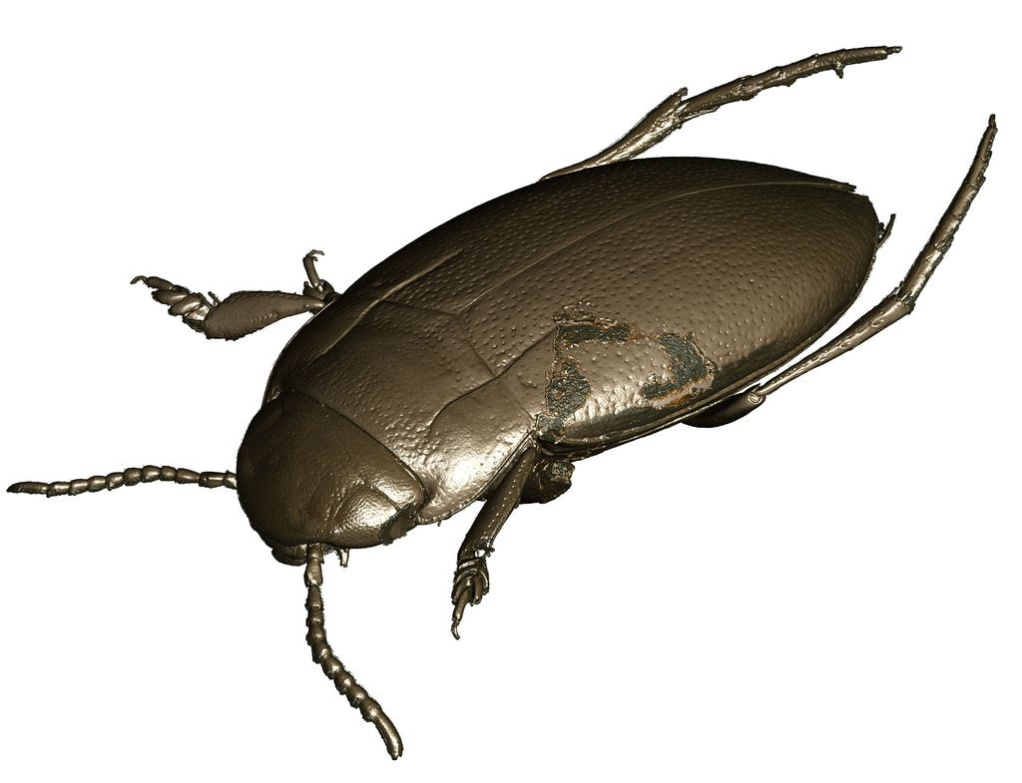
*Limbodessus baliem*, male, habitus frontally. μCT scan. Length of beetle: 2.8 mm.

**Figure 20.**
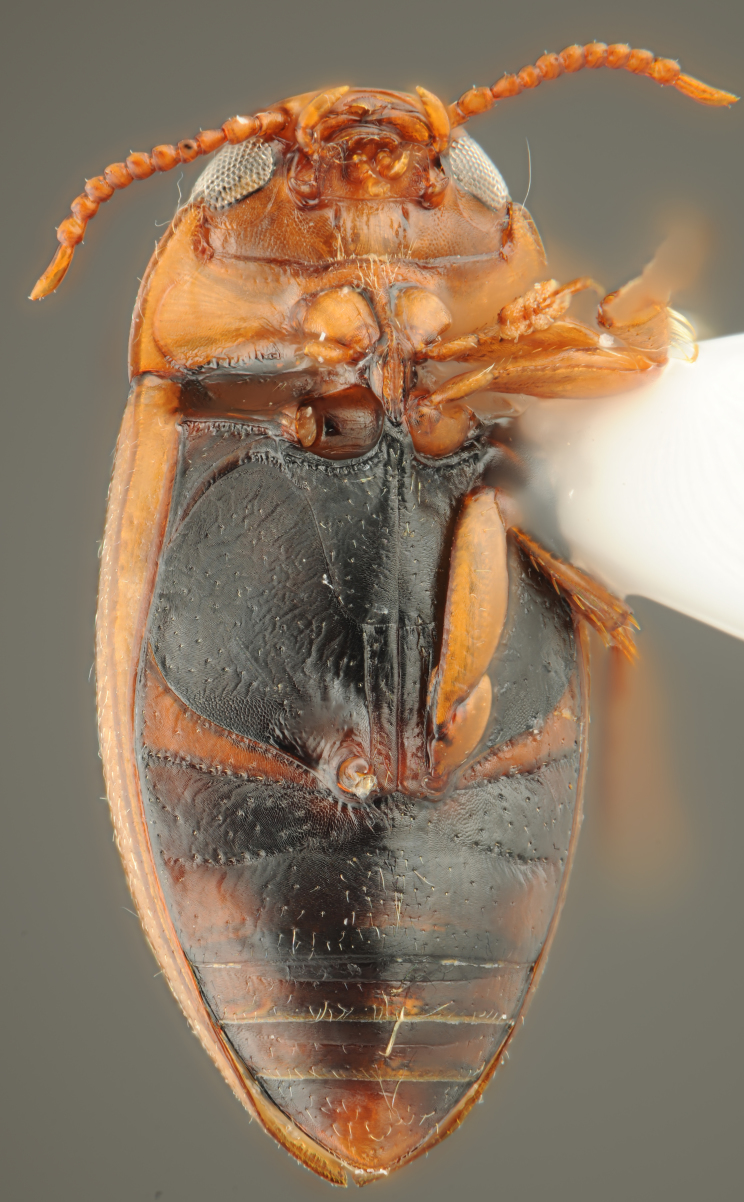
*Limbodessus baliem*, female, habitus ventrally. Length of beetle: 2.8 mm.

**Figure 22.**
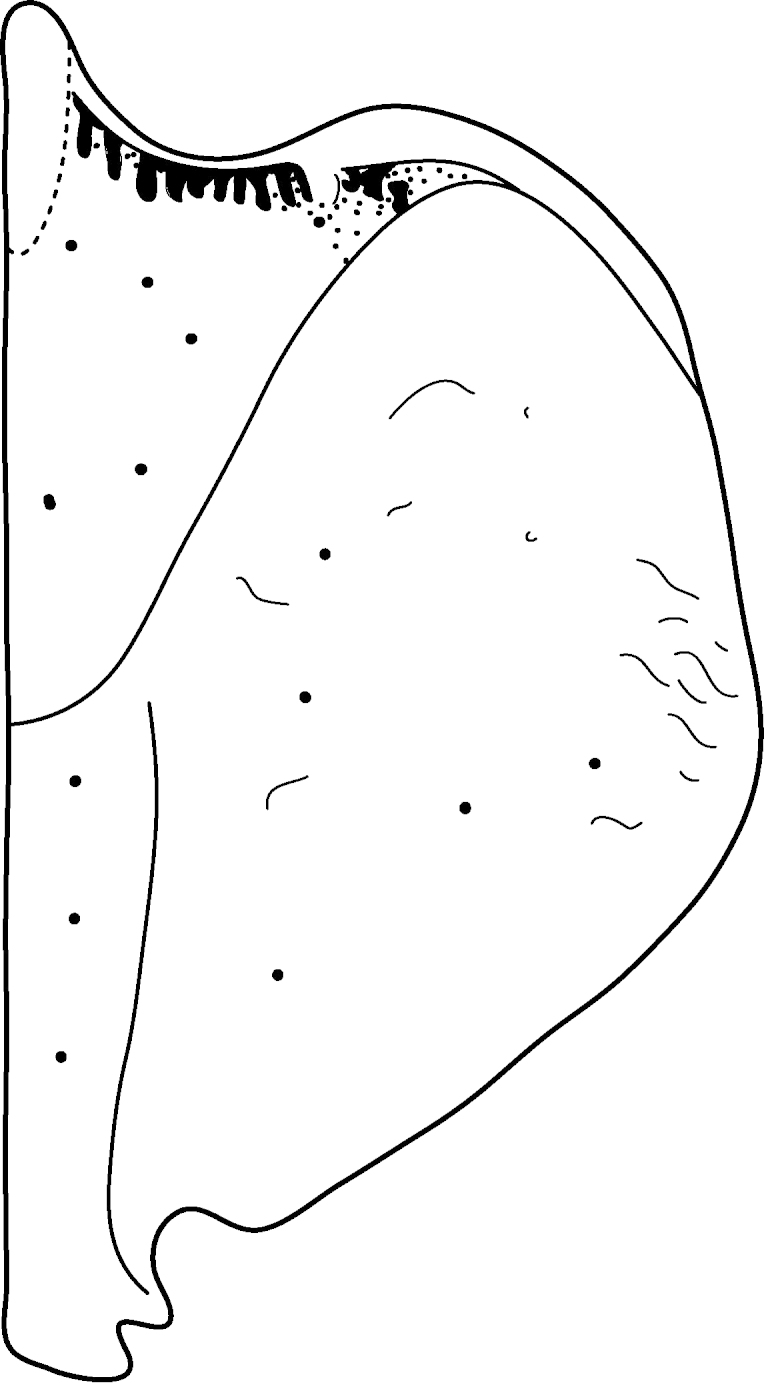
*Limbodessus alexanderi*, ventral aspect, metacoxa and metaventrite.

Antenna moniliform Fig. 14. Dorsal surface with very fine microreticulation between surface punctures and surfaces therefore appearing matt Figs 12, 14. Ventrally matt, darker, metacoxa and metaventrite blackish Fig. 20​.

#### Etymology

The species is named after the type locality, the Baliem River Valley. The name is a noun in the nominative singular standing in apposition.

#### Distribution

**Figure 23.**
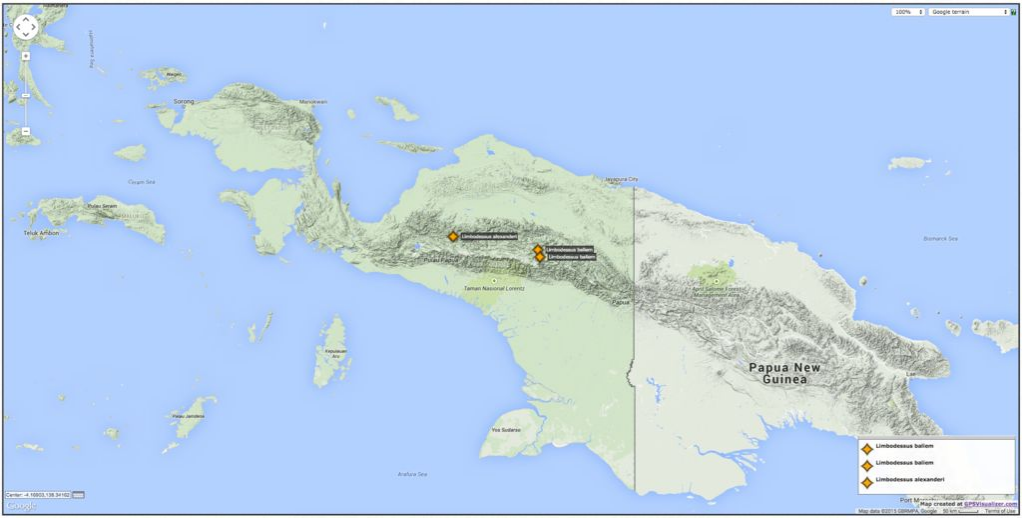
Distribution of *Limbodessus baliem* and *L. alexanderi* in New Guinea. *Limbodessus compactus* is widespread especially in the lowlands, it is absent from the Baliem Valley and altitudes above 2000 m.

**Figure 25.**
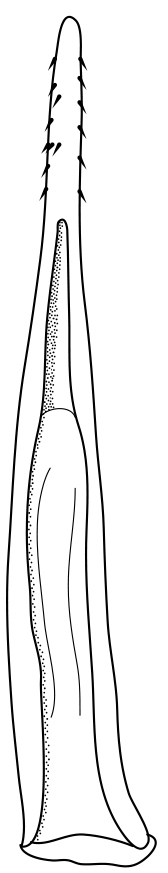
*Limbodessus alexanderi*, male, median lobe in ventral view.

**Figure 26.**
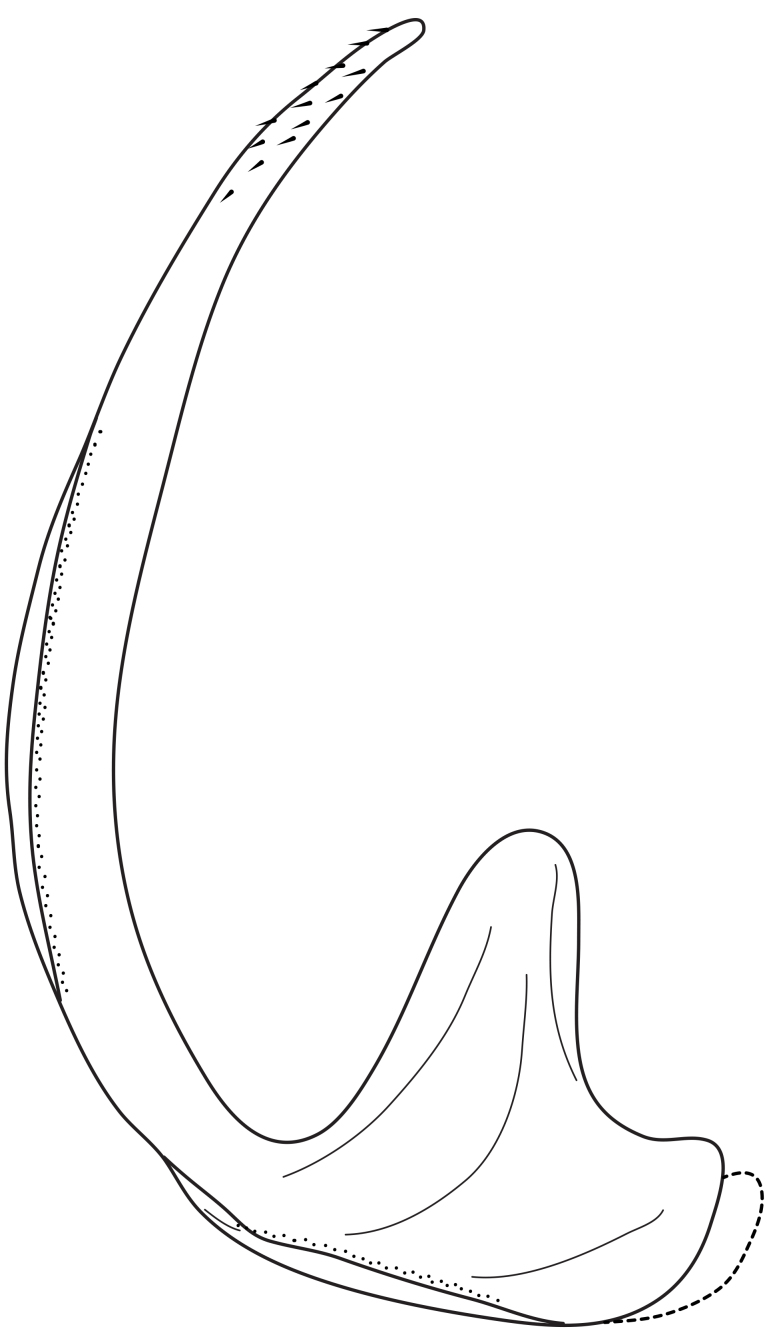
*Limbodessus alexanderi*, male, median lobe in lateral view.

**Figure 27.**
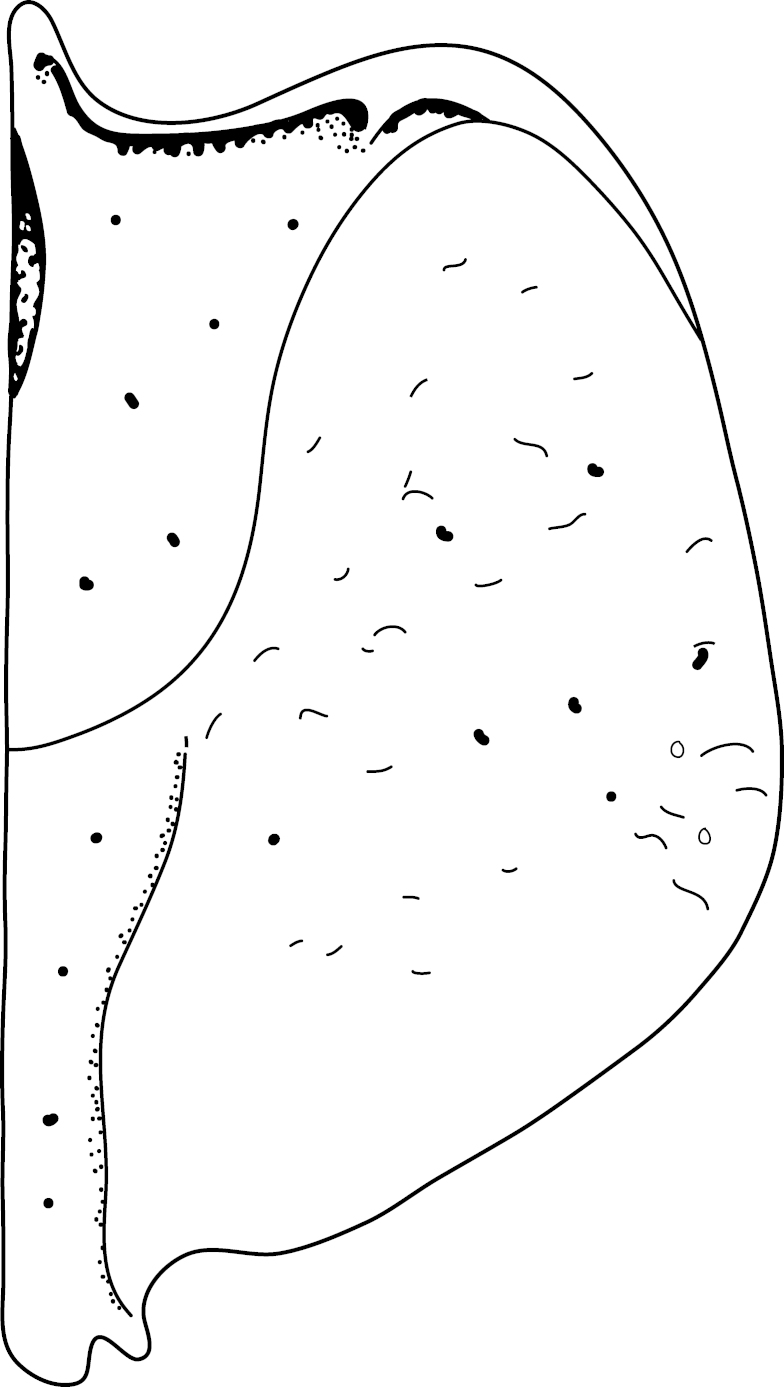
*Limbodessus baliem*, ventral aspect, metacoxa and metaventrite.

**Figure 31.**
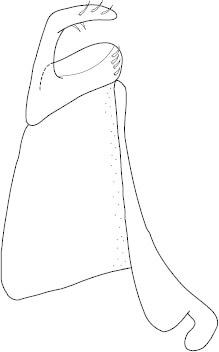
*Limbodessus alexanderi*, male, paramere. Total length 0.46 mm.

Indonesian New Guinea, known only from the Baliem Valley which also contains the type locality Wamena (Fig. 23​). The name is a noun in the nominative singular standing in apposition.

#### Ecology

The species was collected from small bodies of stagnant water around Wamena in the vast, flat valley floor of the Baliem Valley Figs 42, 43​. This species seems to prefer sunny or partly shaded habitat with sand or clay on the bottom and not too strongly vegetated. Other species it was associated with were: *Exocelina baliem* Shaverdo, Hendrich & Balke, 2013, *Hydrovatus enigmaticus* Biström, 1997, *Hyphydrus dani* Biström, Balke & Hendrich, 1993, *Hydaticus okalehubyi* Balke & Hendrich, 1992, *H. rivanolis* Wewalka, 1979, and *Rhantus dani* Balke, 2001 (Shaverdo et al. (2013), Biström et al. (1993), Balke and Hendrich (1992), Balke (2001).

#### Conservation

Aquatic habitats in the Baliem Valley in general are threatened by eutrophication from domestic animals, fish farming to some degree and increasing intensity of gardening throughout the valley.

#### Online resources

Higher resolution digital images, μCT data as well as sequence data have been deposited in public databases. The Species-ID species page is a versioned wiki site and can be enhanced through community contributions Hendrich and Balke (2011)​.

##### Species-ID species page

http://species-id.net/wiki/***Limbodessus_baliem***

##### DNA sequences

Partial 3' *cox1* sequence deposited at: LN884313–LN884314.

##### μCT data

Deposited in Morphosource.

## Identification Keys

### Key to New Guinea *Limbodessus*

**Table:** 

1	Base of elytral epipleuron with raised transverse carina delineating a basal pit, see Fig. 36 (widespread in the Australian region; ventral aspect as in Fig. 28, with metaventrite and metacoxa rather smooth; male genital as in Figs 32, 33, 35).	*Limbodessus compactus* (Clark, 1862)
–	Base of elytral epipleuron without raised transverse carina delineating a basal pit as in Fig. 38​	2
2	Elytral plica present; beetle mainly yellowish; microendemic of the Baliem Valley of Papua	*Limbodessus baliem* **sp.n.**
–	Elytral plica absent; beetle dark brown to blackish; only known from high altitude grassland of the range north of Sugapa, Papua	*Limbodessus alexanderi* **sp.n.**

Based on Watts and Leijs (2005)​.

## Analysis

**Figure 40.**
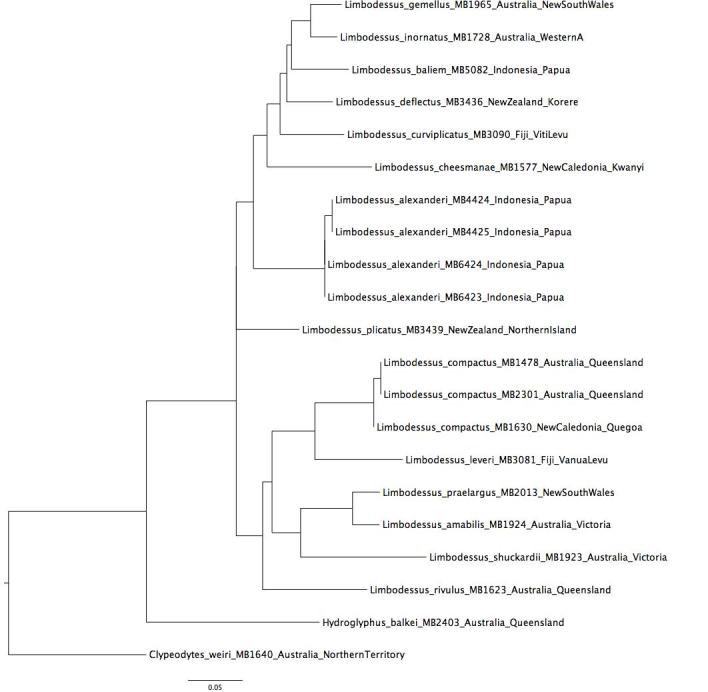
Neighbour joining tree using 3' cox1 sequence data for New Guinea *Limbodessus* with other epigean species from neighbouring areas. *Limbodessus capeensis* Watts and Leijs (2005) was not available.

**Figure 42.**
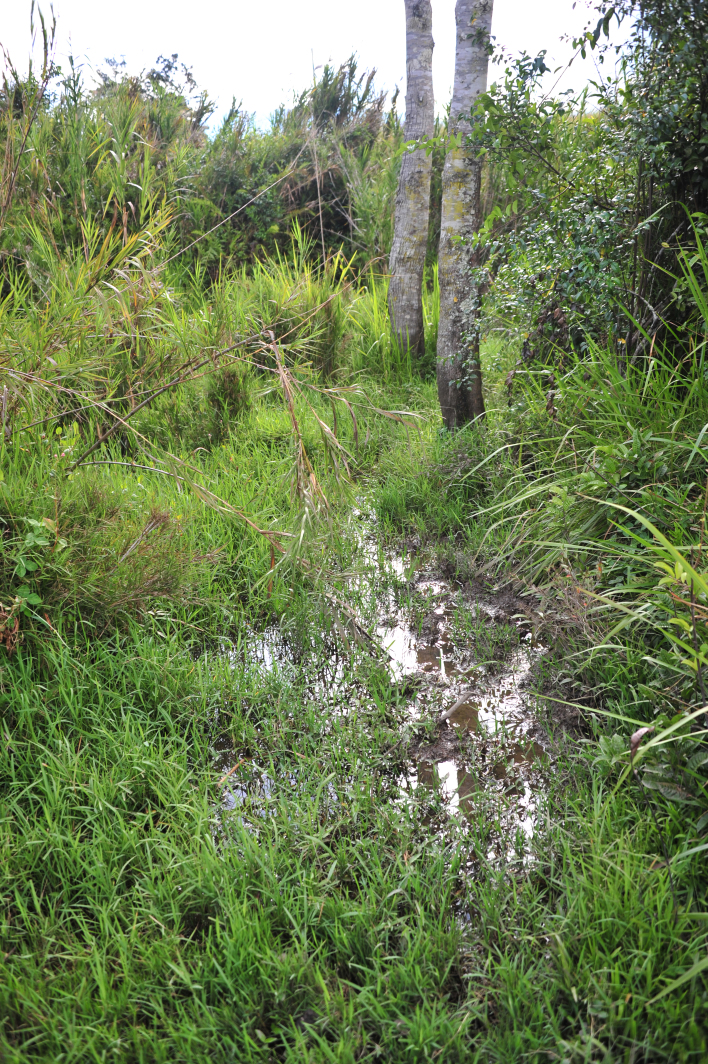
Habitat of *Limbodessus baliem* near Wamena.

**Figure 43.**
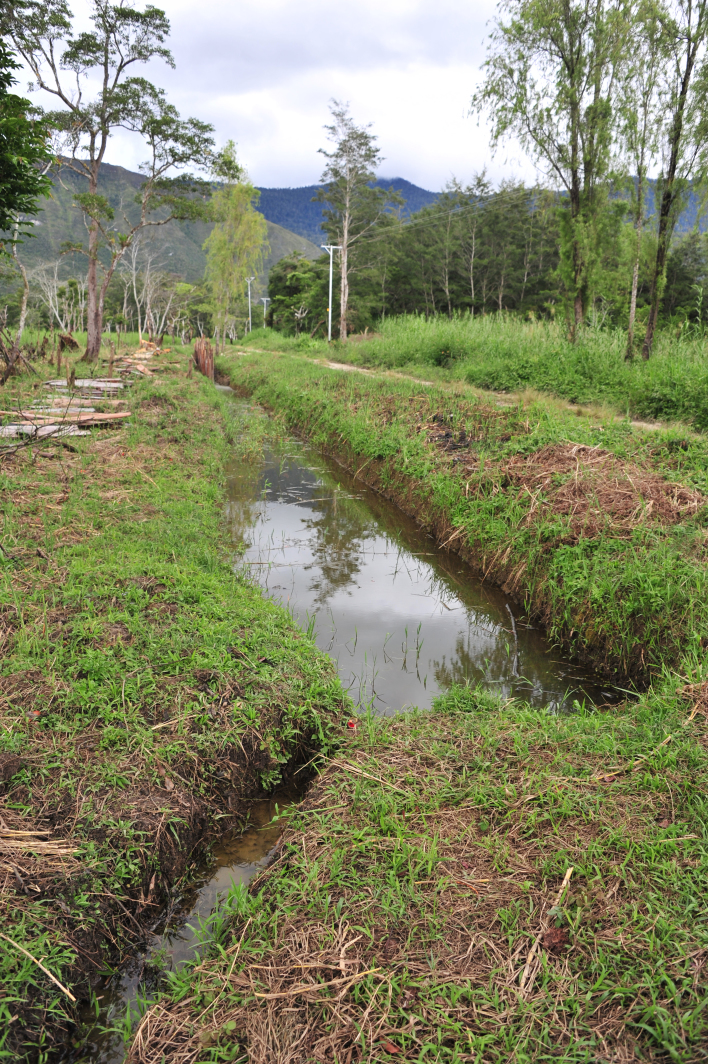
Habitat of *Limbodessus baliem* near Jiwika.

We obtained sequence data for the 3' end of the mitochondrial cytochrome oxidase I gene and analyzed these in the context of our database of Australasian diving beetles Hendrich et al. (2010) by constructing neighbour joining trees (HKY model as implemented in Geneious 8.1.6 software) as well as by clustering the data in SpeciesIdentifier module of Taxon DNA software (v1.6.; Meier et al. (2006). Both species described here differ from known extant taxa (Fig. 40). We queried the two new species against the other *Limbodessus* in our dataset and found uncorrected *p*-distances for *L. baliem* sp.n. of 6.68–12.23% (*L. gemellus* (Clark 1862)/ *L. praelargus* (Lea 1899)) and for *L. alexanderi* sp.n. of 8.83–13.96% (*L. inornatus* (Sharp 1882)/ *L. cheesmanae* (Balfour-Browne 1939)).

As another result of our analysis, we propose new combinations for the following three species:

*Limbodessus deflectus* (Ordish 1966), **new combination**

- *Liodessus deflectus* Ordish 1966: 229; Nilsson 2015: 115.

*Limbodessus leveri* (Balfour-Browne 1944), **new combination**

- *Bidessus leveri* Balfour-Browne 1944: 98; Nilsson 2015: 126.

*Limbodessus plicatus* (Sharp 1882), **new combination**

- *Bidessus plicatus* Sharp 1882: 360

- *Liodessus plicatus* (Sharp 1882): Nilsson 2015: 116.

## Discussion

The merits and possible applications of (X-ray) Micro-CT for arthropod systematics have recently been discussed by Friedrich et al. (2013) and Simonsen and Kitching (2014). The strength of this approach lies in the non-destructive examination. Data acquisition is relatively straight forward and data cover a wide range of information including that of internal structures.

In our examples the CT examination provided comprehensive information on overall morphology; general proportions as well as external surface details could be assessed. Regarding surface details, Micro-CT complements conventional ways of examinations, such as light microscopic photography and SEM. Both methods are superior concerning structural surface details. In addition, light microscopy provides true colours. In the specimens examined in the present study, the X-ray absorption was relatively poor, this might be the reason for some deficiencies in structural resolution. In places, internal structures cannot be discerned. For example, the elytra could not be separated from the underlying material.

The external surface renderings appear to be a very useful approach for distributing the CT data. By interactive manipulation, these polygonal mesh surfaces enable intuitive understanding of overall proportions of the specimens. They also contain an enormous amount of geometric information that could easily be extracted and used for e.g., morphometric studies. The 3D PDF models make the entire external morphological information available to “readers”, who might use this information in further comparative studies.

In order to also provide information on specimen coloration and surface microsculpture, we provided digital photographs of different aspects of the beetles using a high throughput, automated imaging approach that could easily be implemented by a technician. The goal was to optimize image quality and time spent on each image so that taxonomically relevant or potentially interesting details can be recognized easily. Digital imaging does, in our opinion, still outperform CT examination for routine work and in terms of documentation of taxonomically relevant structures in the beetles studied here.

## Supplementary Material

Supplementary material 1 Limbodessus Papua sequences fasta file Data type: DNA sequences
Brief description: A fasta file containing the cox1 sequences newly generated for this project.
File: oo_58458.pdf Michael balke

XML Treatment for Limbodessus alexanderi

XML Treatment for Limbodessus baliem

## Figures and Tables

**Figure 19.**
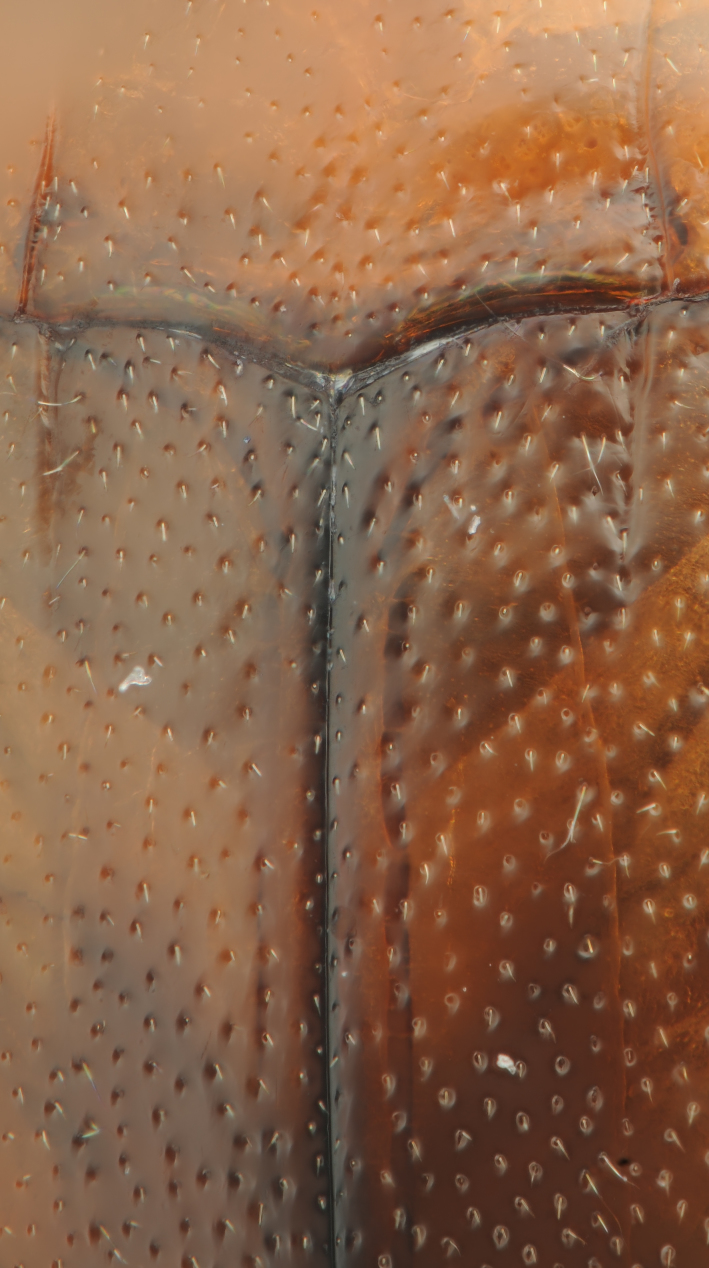
*Limbodessus baliem*, male, detailed elytral and pronotal sculpture. Lens: Mitutoyo ELWD 10x.

**Figure 21.**
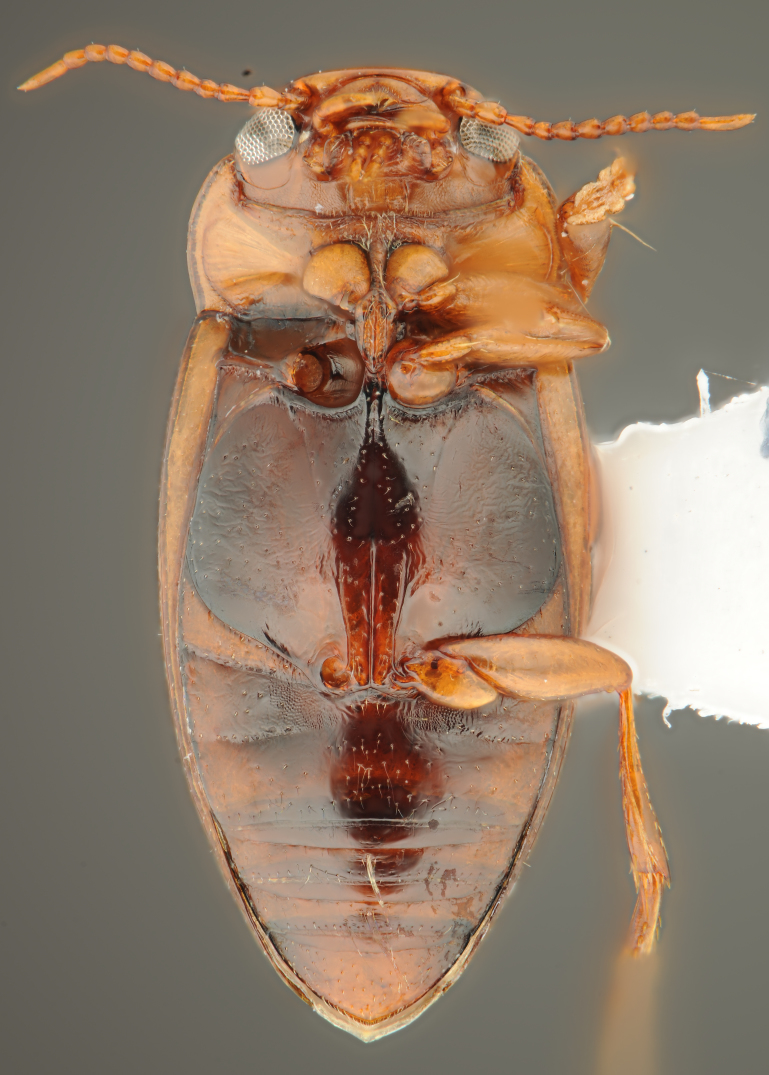
*Limbodessus baliem*, male, habitus ventrally. Length of beetle: 2.8 mm.

**Figure 24.**
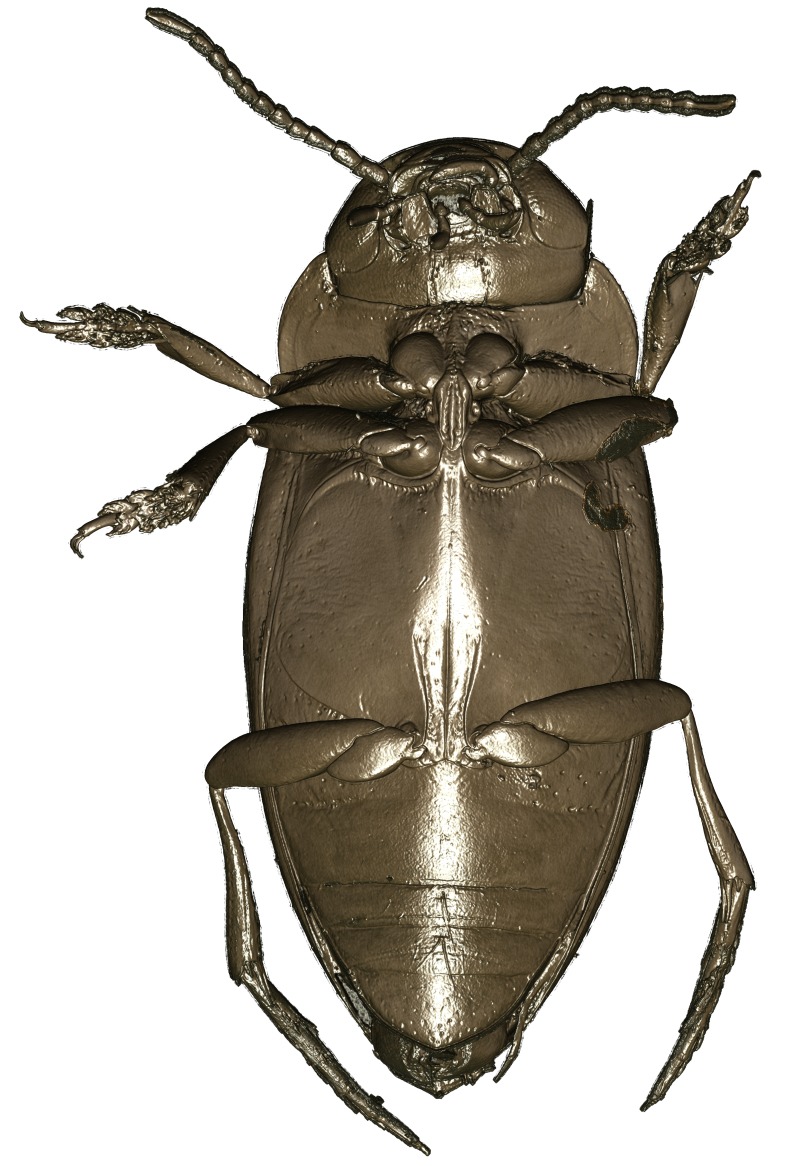
*Limbodessus baliem*, male, habitus ventrally. μCT scan. Length of beetle: 2.8 mm.

**Figure 28.**
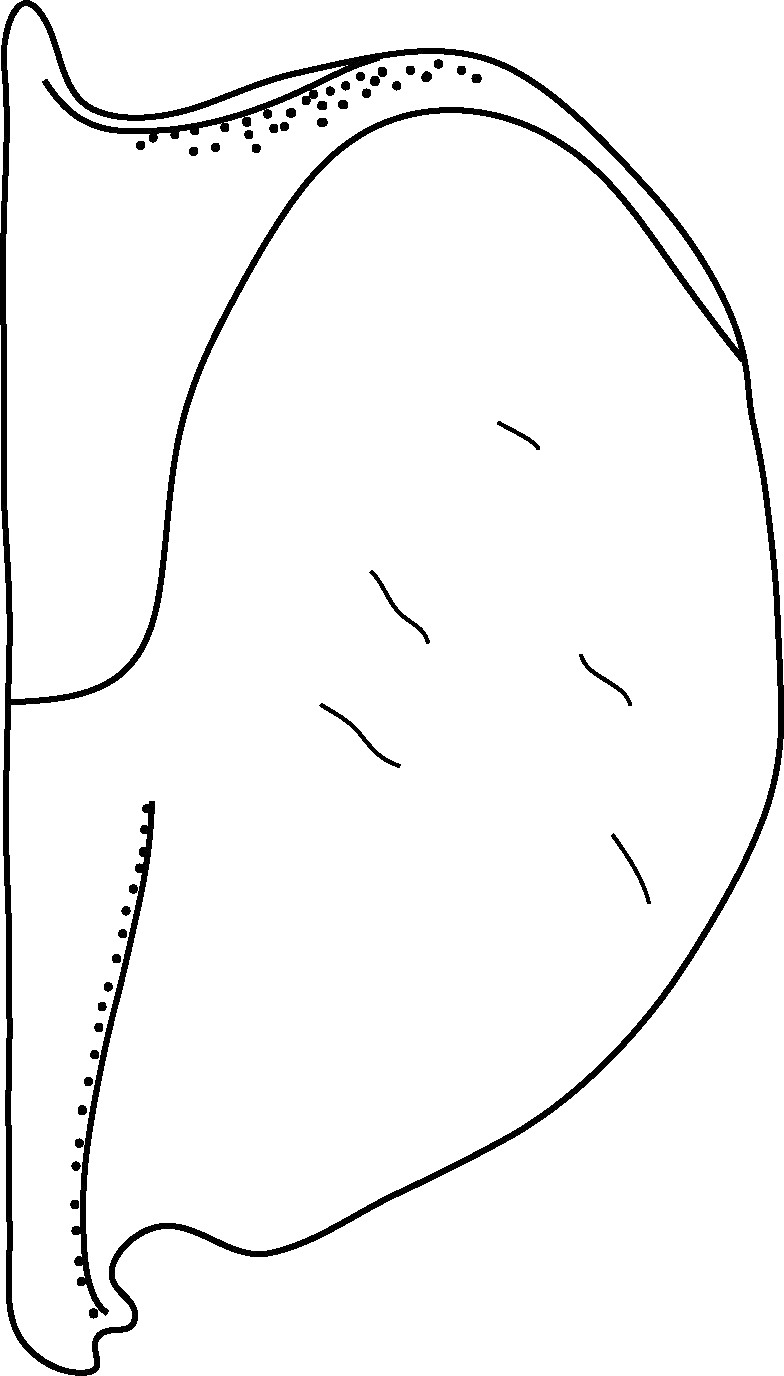
*Limbodessus compactus*, ventral aspect, metacoxa and metaventrite.

**Figure 29.**
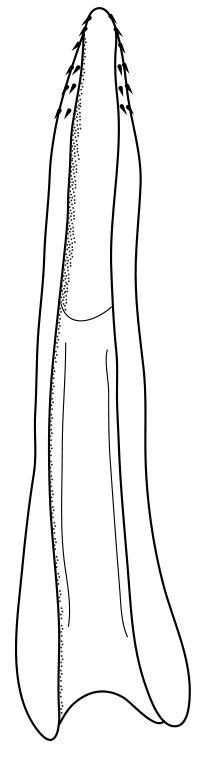
*Limbodessus baliem*, male, median lobe in ventral view.

**Figure 30.**
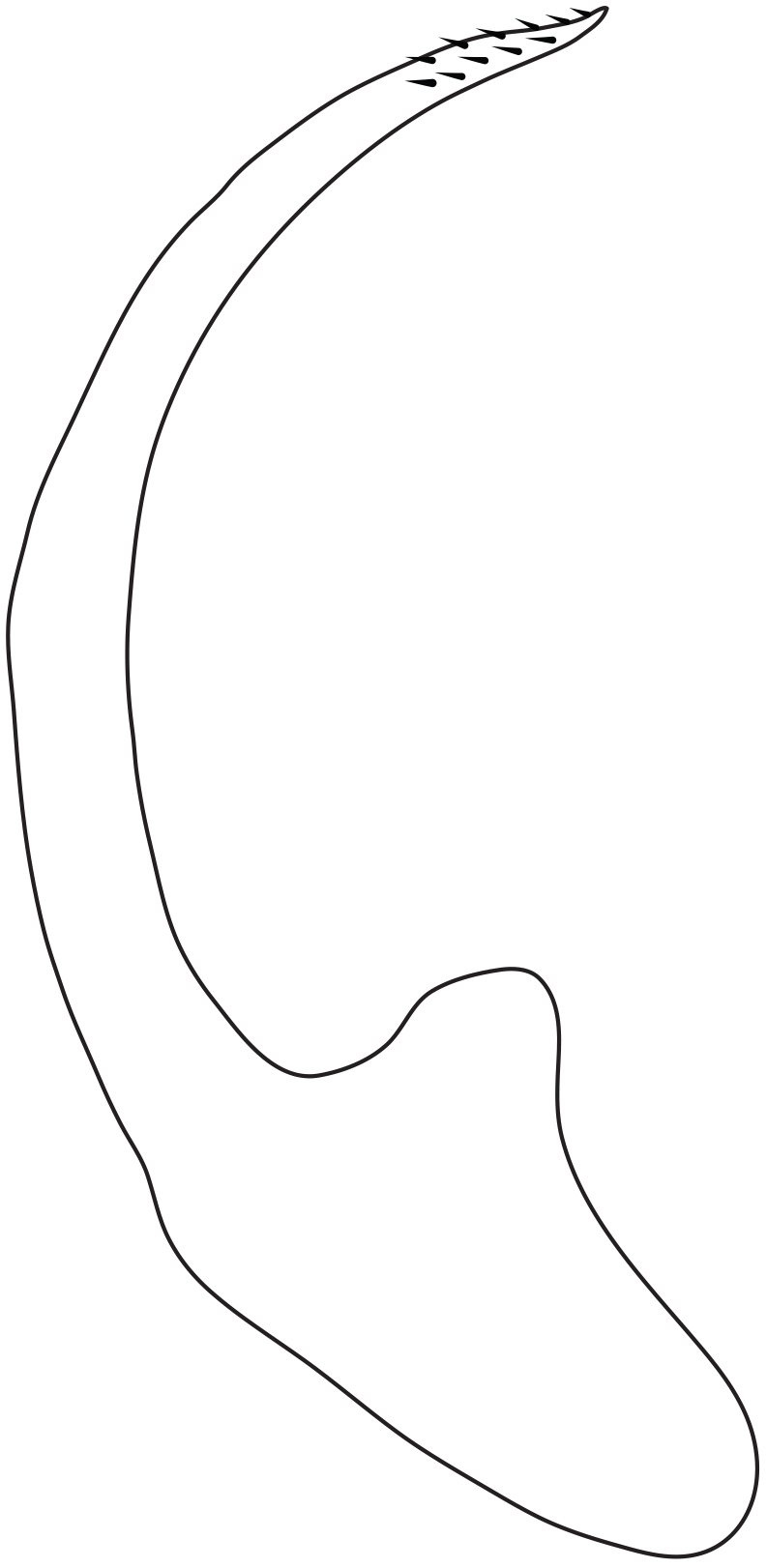
*Limbodessus baliem*, male, median lobe in lateral view.

**Figure 32.**
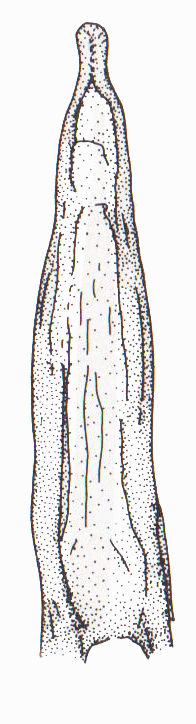
*Limbodessus compactus*, male, median lobe in ventral view (after Biström 1988).

**Figure 33.**
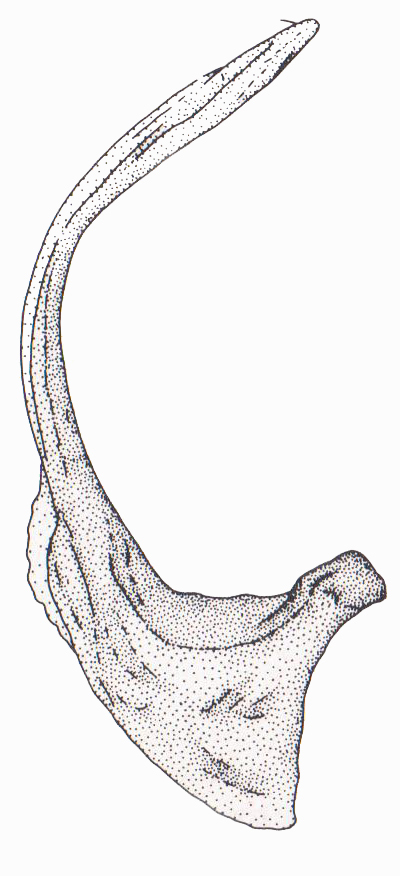
*Limbodessus compactus*, male, median lobe in lateral view (after Biström, 1988).

**Figure 34.**
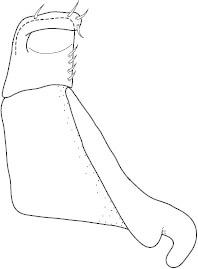
*Limbodessus baliem*, male, paramere. Total length 0.33 mm.

**Figure 35.**
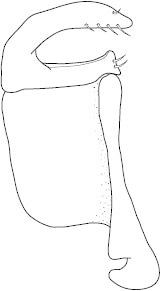
*Limbodessus compactus*, male, paramere. Total length 0.3 mm

**Figure 36.**
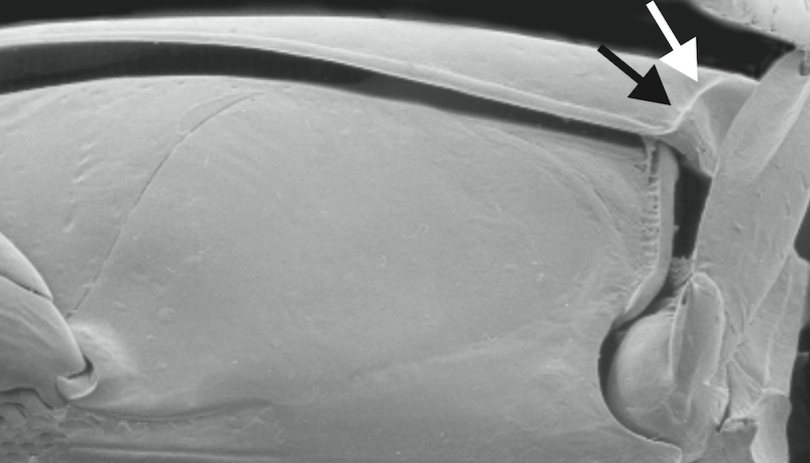
Elytral epipleuron of *Limbodessus compactus*, arrows point to base where there is a carina.

**Figure 37.** *Limbodessus alexanderi*, male, habitus. Animated μCT scan. Length of beetle: 3.1 mm.

**Figure 38.**
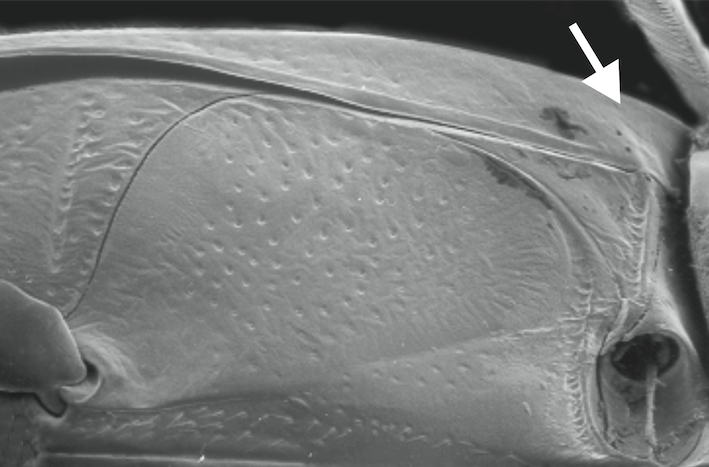
Elytral epipleuron of *Allodessus megacephalus* (Gschwendtner 1931​), arrow points to base where there is no carina.

**Figure 39.** *Limbodessus alexanderi*, female, habitus. Animated μCT scan. Length of beetle: 3.1 mm.

**Figure 41.** *Limbodessus baliem*, female, habitus. Animated μCT scan. Length of beetle: 2.8 mm.
