# Supplementary material for: Two new species of Limbodessus diving beetles from New Guinea - short verbal descriptions flanked by online content (digital photography, μCT scans, drawings and DNA sequence data)
Source: Biodivers Data J. 2015 Dec 22;(3):e7096. doi: 10.3897/BDJ.3.e7096 (PMC4700388; doi:10.3897/BDJ.3.e7096)
Supplement: Supplementary material 1 — Limbodessus Papua sequences fasta file [file biodiversity_data_journal-3-e7096-s001.pdf]

```
>Limbodessus plicatus; MB3439; NewZealand; NorthernIsland;
ATTATTAGTCAAGAAAGAGGAAAAAAGGAAACATTTGGATCATTGGGGATAATCTATGCTATACTAGCTATTGGGTTATT
GGGATTTGTAGTGTGAGCTCATCATATATTCACGGTAGGAATAGATGTTGATACACGAGCATATTTTACCTCAGCAACTA
TAATTATTGCAGTCCCAACAGGAATTAAAATTTTTTCTTGATTAGCAACCTTACATGGTTCTCAAATATCTTATAGACCA
TCACTGTTATGAGCATTAGGGTTTGTATTTTTATTTACTGTTGGGGGTTTAACAGGAGTAGTATTGGCCAATTCTTCTAT
TGATATTATTCTTCATGATACATATTATGTAGTAGCTCATTTTCATTATGTTTTATCAATAGGGGCTGTATTTGCTATTT
TAGGAGGATTTATTCAATGATTCCCCCTATTTACTGGATTATCCCTAAATTCTAATTTACTAAAAACTCAATTTATTATT
ATATTTATTGGAGTAAATCTTACATTCTTTCCTCAACATTTTTTAGGACTAAGAGGAATACCTCGACGATATTCTGATTA
TCCAGATGCATATACCCCCTGAAATGTAATCTCTTCAATAGGATCAACTATTTCATTTATTGGGGTATTAATATTTATTT
ATATTATTTGAGAAGCTTTTATTGCTCAACGATTAGTAATTTTCTCTAATAGAATATCAACTTCAATTGAGTGATTCCAA
CTTTTCCCACCATCTG
>Limbodessus leveri; MB3081; Fiji; VanuaLevu;
TTTGGGTCATTAGGAATAATTTATGCTATATTAGCTATTGGATTATTAGGATTTGTTGTCTGAGCTCATCACATATTTAC
AGTAGGAATAGATGTCGATACACGAGCATATTTCACATCAGCAACTATAATTATTGCAGTTCCTACCGGAATTAAAATTT
TCTCTTGATTAGCAACATTACATGGTTCTCAAATATCTTATAGCCCATCATTATTATGAGCACTGGGATTTGTTTTTTTA
TTTACAGTAGGGGGTTTAACAGGGGTGGTACTAGCTAACTCTTCAATTGATATTATTCTACATGATACTTATTATGTTGT
AGCTCATTTTCACTACGTTTTATCAATAGGAGCTGTGTTCGCTATTTTGGGTGGATTTATTCAATGATTTCCCCTATTTA
CTGGTTTATCTTTAAATTCTAACTTATTAAAAACACAATTCATTATTATATTTGTGGGAGTTAATTTAACATTTTTTCCC
CAACATTTCTTAGGATTAAGAGGAATACCCCGACGATACTCTGATTACCCGGATGCATATACTTCCTGAAATGTAATCTC
TTCCATAGGATCAACTATTTCATTTATTGGAGTATTGATATTTATTTACATTATTTGAGAAGCATTTATTGCTCAACGAT
TAGTAGTTTTCTCTAATAGAATGTCAACCTCAATTGAATGATTCCAACTCTTCCCCCCATCTG
>Limbodessus deflectus; MB3436; NewZealand; Korere;
ATTATTAGTCAAGAAAGAGGAAAAAAAGAAACATTCGGATCATTAGGAATAATTTATGCTATACTAGCTATTGGATTATT
GGGATTTGTAGTATGAGCTCACCATATATTTACAGTAGGAATAGATGTTGATACGCGAGCATATTTCACATCAGCAACTA
TAATTATTGCAGTACCAACAGGGATTAAAATTTTCTCTTGGTTAGCAACTTTACATGGTTCTCAAATATCTTATAGACCA
TCTTTACTATGAGCATTGGGGTTTGTATTCTTATTTACTGTGGGGGGGTTAACGGGGGTAGTTTTAGCTAATTCATCTAT
TGATATTATTCTTCACGATACCTATTATGTAGTGGCTCATTTCCATTACGTTTTATCAATAGGGGCTGTATTTGCTATTT
TAGGGGGATTTATTCAATGATTTCCCCTATTTACTGGGTTATCTTTAAACTCAAACTTATTAAAAACCCAATTTATTATT
ATATTTATTGGGGTAAATTTAACATTCTTCCCTCAACATTTCTTAGGATTAAGAGGTATACCTCGACGATATTCCGATTA
TCCAGATGCATATACTTCATGAAATATTATTTCCTCAATAGGATCAACTATTTCATTTATTGGAGTATTAATATTTATTT
ATATTATTTGAGAAGCATTTATTACCCAACGATTAGTGATTTTCTCTAATAGTATATCAACCTCAATTGAATGATTCCAA
CTTTTCCCACCTTCTG
>Limbodessus curviplicatus; MB3090; Fiji; VitiLevu;
ATTATTAGTCAAGAAAGAGGAAAAAAGGAGACATTTGGATCACTAGGAATAATTTATGCTATACTAGCTATTGGGTTATT
AGGATTTGTAGTATGAGCTCATCATATATTTACAGTAGGAATAGATGTTGATACACGAGCATATTTTACATCAGCAACTA
TAATTATTGCCGTACCAACAGGAATTAAAATTTTCTCTTGATTAGCAACTTTACATGGTTCTCAAATATCTTATAGACCA
TCTTTACTGTGAGCCTTAGGGTTTGTATTTTTATTTACTGTTGGAGGTTTAACGGGGGTAGTATTGGCTAATTCATCTAT
TGATATTATCCTTCACGATACATACTATGTAGTAGCCCATTTCCATTACGTTTTATCAATAGGAGCTGTATTTGCTATTC
TAGGAGGATTTATTCAATGATTTCCTTTATTTACTGGGTTATCATTAAACTCAAATTTATTAAAAACTCAATTTATTGTT
ATATTTATTGGAGTAAATTTAACATTTTTTCCTCAACATTTCTTAGGATTAAGAGGAATACCTCGACGATATTCTGATTA
TCCAGACGCATATACTTCATGAAATGTAATCTCCTCAATAGGATCAACTATTTCATTTATTGGAGTATTAATATTTATTT
ATATTATCTGAGAAGCATTTATTGCTCAACGATTAGTAATTTTCTCTAATAATATATCAACCTCAATTGAATGATTCCAA
CTTTTTCCACCCTCTG
>Limbodessus newspeciesDorman; MB4424; Indonesia; Papua;
CGACGATATTCTGATTATCCAGAGGCATATACATGTTGAAATGTAATTTCCTCAGTAGGATCAACTATCTCATTTATTGG
AGTATTAATATTTATYTATATTATTTGAGAAGCTTTTATTGCTCAACGATTAGTAATTTTTTCTAACAGAATATCAACCT
CAATTGAATGATTCCAACTTTTTCCACCCTCTG
>Limbodessus newspeciesDorman; MB4425; Indonesia; Papua;
CGACGATATTCTGATTATCCAGAGGCATATACATGTTGAAATGTAATTTCCTCAGTAGGATCAACTATCTCATTTATTGG
AGTATTAATATTTATYTATATTATTTGAGAAGCTTTTATTGCTCAACGATTAGTAATTTTTTCTAACAGAATATCAACCT
CAATTGAATGATTCCAACTTTTTCCACCCTCTG
>Limbodessus newspeciesDorman; MB6423; Indonesia; Papua;
CGACGATATTCTGATTATCCAGATGCATATACATGTTGAAATGTAATTTCCTCAGTAGGATCAACTATCTCATTTATTGG
AGTATTAATATTTATYTATATTATTTGAGAAGCTTTTATTGCTCAACGATTAGTAATTTTTTCTAACAGAATATCAACCT
CAATTGAATGATTCCAACTTTTTCCACCCTCTG
>Limbodessus newspeciesDorman; MB6424; Indonesia; Papua;
CGACGATATTCTGATTATCCAGATGCATATACATGTTGAAATGTAATTTCCTCAGTAGGATCAACTATCTCATTTATTGG
AGTATTAATATTTATYTATATTATTTGAGAAGCTTTTATTGCTCAACGATTAGTAATTTTTTCTAACAGAATATCAACCT
CAATTGAATGATTCCAACTTTTTCCACCCTCTG
```

>Limbodessus newspeciesBaliem; MB5082; Indonesia; Papua;
ATTATTAGTCAAGAAAGAGGGAAAAAGGAAACATTTGGATCATTGGGGATAATTTATGCTATATTAGCTATTGGATTATT
GGGATTTGTAGTATGAGCTCATCATATATTTACAGTGGGGATAGATGTTGATACACGAGCATATTTTACATCAGCAACTA
TAATTATTGCAGTACCAACAGGAATTAAAATTTTCTCTTGATTGGCAACATTACACGGTTCTCAAATATCTTATAGACCA
TCTTTACTATGGGCATTAGGATTTGTATTTTTATTTACTGTAGGGGGGTTAACGGGGGTGGTATTAGCTAACTCATCTAT
TGATATTATTCTTCATGATACTTATTATGTAGTAGCTCATTTCCATTACGTTTTATCAATGGGGGCCGTATTTGCTATTT
TAGGGGGATTTATCCAATGATTTCCCCTATTCACTGGATTATCCTTAAACTCAAATTTATTAAAAACCCAATTTATTGTT
ATATTTATTGGAGTAAATTTAACATTCTTCCCTCAACATTTCTTAGGATTAAGAGGAATACCTCGACGATATTCTGATTA
TCCAGACGCATATACTTCATGAAATGTGATTTCCTCAATAGGATCAACAATTTCATTTATTGGAGTAATTATATTTATTT
ATATCATTTGAGAAGCTTTTATTGCTCAACGATTAGTAATTTTCTCTAATAGAATGTCAACCTCAGTTGAATGATTCCAA
CTTTTCCCACCCTCTG
>Limbodessus newspeciesBaliem; MB5083; Indonesia; Papua;
ATTATTAGTCAAGAAAGAGGGAAAAAGGAAACATTTGGATCATTGGGGATAATTTATGCTATATTAGCTATTGGATTATT
GGGATTTGTAGTATGAGCTCATCATATATTTACAGTGGGGATAGATGTTGATACACGAGCATATTTTACATCAGCAACTA
TAATTATTGCAGTACCAACAGGAATTAAAATTTTCTCTTGATTGGCAACATTACACGGTTCTCAAATATCTTATAGACCA
TCTTTACTATGGGCATTAGGATTTGTATTTTTATTTACTGTAGGGGGGTTAACGGGGGTGGTATTAGCTAACTCATCTAT
TGATATTATTCTTCATGATACTTATTATGTAGTAGCTCATTTCCATTACGTTTTATCAATGGGGGCCGTATTTGCTATTT
TAGGGGGATTTATCCAATGATTTCCCCTATTCACTGGATTATCCTTAAACTCAAATTTATTAAAAACCCAATTTATTGTT
ATATTTATTGGAGTAAATTTAACATTCTTCCCTCAACATTTCTTAGGATTAAGAGGAATACCTCGACGATATTCTGATTA
TCCAGACGCATATACTTCATGAAATGTGATTTCCTCAATAGGATCAACAATTTCATTTATTGGAGTAATTATATTTATTT
ATATCATTTGAGAAGCTTTTATTGCTCAACGATTAGTAATTTTCTCTAATAGAATGTCAACCTCAGTTGAATGATTCCAA
CTTTTCCCACCCTCTG
